# Green-aware intrusion detection systems: A sustainability-driven approach to network security

**DOI:** 10.1371/journal.pone.0355453

**Published:** 2026-10-15

**Authors:** Abdullah Alshammari

**Affiliations:** College of Computer Science and Engineering, University of Hafr Albatin, Hafar Albatin, Saudi Arabia; Maulana Abul Kalam Azad University of Technology West Bengal, INDIA

## Abstract

The rapid proliferation of Internet of Things (IoT) devices has intensified network security challenges. Simultaneously, growing computational demands raise critical concerns about energy consumption and environmental sustainability. This paper proposes a Green-Aware Intrusion Detection System (GA-IDS) integrating sustainability metrics with machine learning to balance detection performance and energy efficiency. The framework employs a lightweight convolutional neural network optimized for resource-constrained platforms with dynamic power management and adaptive computational scaling. A novel carbon-conscious scheduling algorithm dynamically adjusts model complexity based on real-time grid carbon intensity. Experimental evaluation on CICIDS2017, NSL-KDD, and ToN-IoT datasets demonstrates 98.7% detection accuracy with 43% energy reduction compared to traditional deep learning-based IDS. The carbon-aware scheduler reduces emissions by 58.7% during high-carbon grid conditions without compromising detection quality. Comprehensive ablation studies validate component contributions, and comparative analysis against ten state-of-the-art approaches confirms GA-IDS achieves the highest Green Efficiency Score (0.0796), demonstrating superior balance between security performance and environmental responsibility.

## 1. Introduction

The accelerated growth in the number of related devices in the Internet of Things (IoT) ecosystem has fundamentally altered the modern digital infrastructure, providing the possibility of extensive automation, real-time data collection, and decision-making practices grounded in data in major areas of smart cities, industrial control systems, health care, and intelligent transportation. Nevertheless, the ubiquitous interconnectedness has greatly expanded the area of attacks on modern networks, and they are becoming more susceptible to advanced cybercrime. Consequently, Intrusion Detection Systems (IDS) have become established network security requirements, continuously evolving to detect advanced attack signatures within large-volume and heterogeneous traffic environments [1,2].

Over the last few years deep learning based IDS have recorded impressive gains in their detection precision through convolutional architectures, recurrent architectures, and attention architectures. These models are very effective however they have large computational and energy requirements that result in high power consumption and carbon emission at training and deployment [3]. The scale security facilities that are implemented in data centers and edge-cloud systems already consume about 1–2% of the electricity usage globally, and this figure is projected to grow as security analytics expand [4]. This trend creates increasing tension between the necessity of a strong, real-time cybersecurity, on the one hand, and international sustainability, goals, on the other hand, as organizations start adhering to carbon neutrality and environmentally sustainable computing practices [5].

Green computing has proven itself as a potential paradigm towards alleviating the environmental impact of the computational systems focusing on energy efficiency, dynamic use of resources and carbon-conscious operation [6]. Simultaneously, the cybersecurity community has also discovered lightweight intrusion detection schemes, such as the side-channel-based anomaly detectors [7], the entropy-based multivariate time-series analysis feature aggregation schemes [8], and the efficient deep learning architecture for resource-constrained IoT settings [9,10]. The federated and distributed learning models incorporate the additional benefit of limiting the centralised computation and communication overhead, and the data privacy is ensured [11,12]. Even though such studies enhance efficiency, they mostly consider energy use as a secondary optimization goal and fail to explicitly consider the carbon intensity of underlying infrastructural power.

In more recent times, carbon-conscious and low-carbon computer policies have been explored as in the areas of energy systems optimization, smart grids, and distributed task distribution [13,14]. These strategies show that in real time changing the computational workload can greatly lower the amount of emissions without compromising on performance through dynamically adjusting the computational workload based on the current carbon intensity values in the real time grids. Nevertheless, these carbon-conscious mechanisms have not been introduced into intrusion detection systems in a systematic manner, since they usually are latency-sensitive systems with performance under strong reliability thresholds. The current IDS studies thus fail to have a single framework that can optimize detection efficiency, energy efficiency as well as carbon footprint.

Motivation**:** Although there is high advancement in the accuracy of intrusion detection, current IDS models mostly optimize the security performance of the systems as they stand alone and use consumption of energy and effect on the environment as the secondary concerns. Most lightweight and low energy-use detection models are aimed at minimizing the use of computation without necessarily regarding the carbon footprint of the power infrastructure behind it, whereas carbon mindful computing methods are mostly oriented towards deferrable or non-critical loads. However, intrusion detection is relatively restrictive in both latency and reliability and there is no direct implication of suitable techniques. This gap is the driving force behind the need to have an IDS framework that balances detection performance with environmental sustainability. Recent surveys on green computing and sustainable AI [15,16] identify energy-efficient intrusion detection as a critical research direction, while studies on carbon-aware computing [17] emphasize the need for real-time adaptation mechanisms in security-critical systems.

### 1.1. Novel contributions and differentiation

GA-IDS differs from existing frameworks through three distinct algorithmic innovations. First, real-time carbon-aware scheduling (Algorithm 1, Eq. 11) dynamically selects among pre-trained complexity levels {M_1_, M_2_,..., ${\text{M}}_{\text{L}}$} based on grid carbon intensity ${\text{C}}_{\text{t}}$, achieving spatial adaptation within sub-10ms latency constraints rather than temporal workload deferral used in traditional carbon-aware computing. Second multi-objective training (Eqs. 18–21) jointly optimizes: detection loss ${\text{L}}_{\text{det}}$ (Eq. 19, weighted binary cross-entropy), energy regularization ${\text{L}}_{\text{energy}}$ (Eq. 20, L1 sparsity + MAC penalty), and carbon-aware regularization ${\text{L}}_{\text{carbon}}=\text{ E}\left[{\text{E}}_{\text{inf}}\cdot \;{\text{C}}_{\text{t}}\right]$ (Eq. 21, expected carbon cost of inference) during gradient descent. Third, the Green Efficiency Score (GES = F1-score/ (${\text{E}}_{\text{inference}}\cdot \;{\text{C}}_{\text{avg}}$)) provides the first standardized metric quantifying security-sustainability trade-offs across heterogeneous deployments. To substantiate these novelty claims, Table 1 provides a systematic comparison of GA-IDS against closely related approaches across seven dimensions.

**Table 1:** Comparison of GA-IDS with Closely Related Studies.

Feature	Energy-Aware DNN [18]	MobileNet-IDS [7]	EfficientNet-IDS [19]	Carbon-Aware Computing (General)	GA-IDS (Proposed)
Real-time detection	Yes	Yes	Yes	No	Yes
Energy optimization	Yes (DVFS/power gating)	Yes (static compression)	Yes (static scaling)	Partial	Yes (dynamic)
Carbon intensity integration	No	No	No	Yes (temporal deferral)	Yes (spatial adaptation)
Runtime model selection	No	No	No	No	Yes
Joint security-energy-carbon loss	No	No	No	No	Yes
Standardized sustainability metric	No	No	No	No	Yes (GES)
Latency-sensitive adaptation	No	No	No	No	Yes (2.1 ms GPU; up to 8.2 ms edge)

GA-IDS is explicitly differentiated from two related paradigms:

- **Carbon-Aware Computing (Temporal Deferral):** Existing carbon-aware methods defer deferrable workloads to low-carbon periods. GA-IDS instead performs spatial adaptation — selecting model complexity in real-time within latency constraints, without deferring security operations.
- **Energy-Efficient IDS (Static Compression):** Lightweight architectures such as MobileNet-IDS and EfficientNet-IDS apply one-time model compression uniformly at all times. Unlike these static approaches, GA-IDS dynamically adjusts computational intensity based on live grid carbon signals, enabling runtime carbon-conscious adaptation that static compression cannot provide.

Quantitative validation of these distinctions is provided in Table 2, where explicit carbon-aware optimization yields 17.2% higher GES compared to the best energy-efficient baseline (MobileNet-IDS, GES = 0.0679), confirming the measurable advantage of joint security-energy-carbon optimization.

**Table 2:** Comprehensive Comparison with State-of-the-Art and Green-Aware IDS Methods (Mean ± Std over 5 Runs).

Method	Category	Accuracy (%)	F1-Score	Energy (mJ)	GES	Carbon-Aware
CNN-IDS [9]	Standard	96.34 ± 0.42	96.29 ± 0.38	34.2 ± 1.1	0.0281	✗
LSTM-IDS [8]	Standard	97.12 ± 0.36	97.11 ± 0.33	28.7 ± 0.9	0.0338	✗
Transformer-IDS [20]	Standard	98.45 ± 0.25	98.44 ± 0.24	52.4 ± 1.6	0.0188	✗
MobileNet-IDS [7]	Energy-Efficient	96.45 ± 0.41	96.45 ± 0.39	14.2 ± 0.5	0.0679	✗
EfficientNet-IDS [19]	Energy-Efficient	97.92 ± 0.30	97.91 ± 0.28	18.9 ± 0.6	0.0518	✗
Energy-Aware DNN [18]	Energy-Aware ML	96.12 ± 0.44	96.05 ± 0.42	22.3 ± 0.8	0.0431	✗
Pruned CNN-IDS	Model Compression	96.78 ± 0.38	96.72 ± 0.36	19.4 ± 0.6	0.0498	✗
Quantized LSTM-IDS	Model Compression	96.89 ± 0.37	96.84 ± 0.35	15.7 ± 0.5	0.0617	✗
GA-IDS (Proposed)	**Green-Aware**	**98.72 ± 0.18**	**98.67 ± 0.16**	**12.4 ± 0.3**	**0.0796**	✓

Fig 1: presents the conceptual framework of how to combine the green computing principles with the intrusion detection systems.

**Fig 1:**
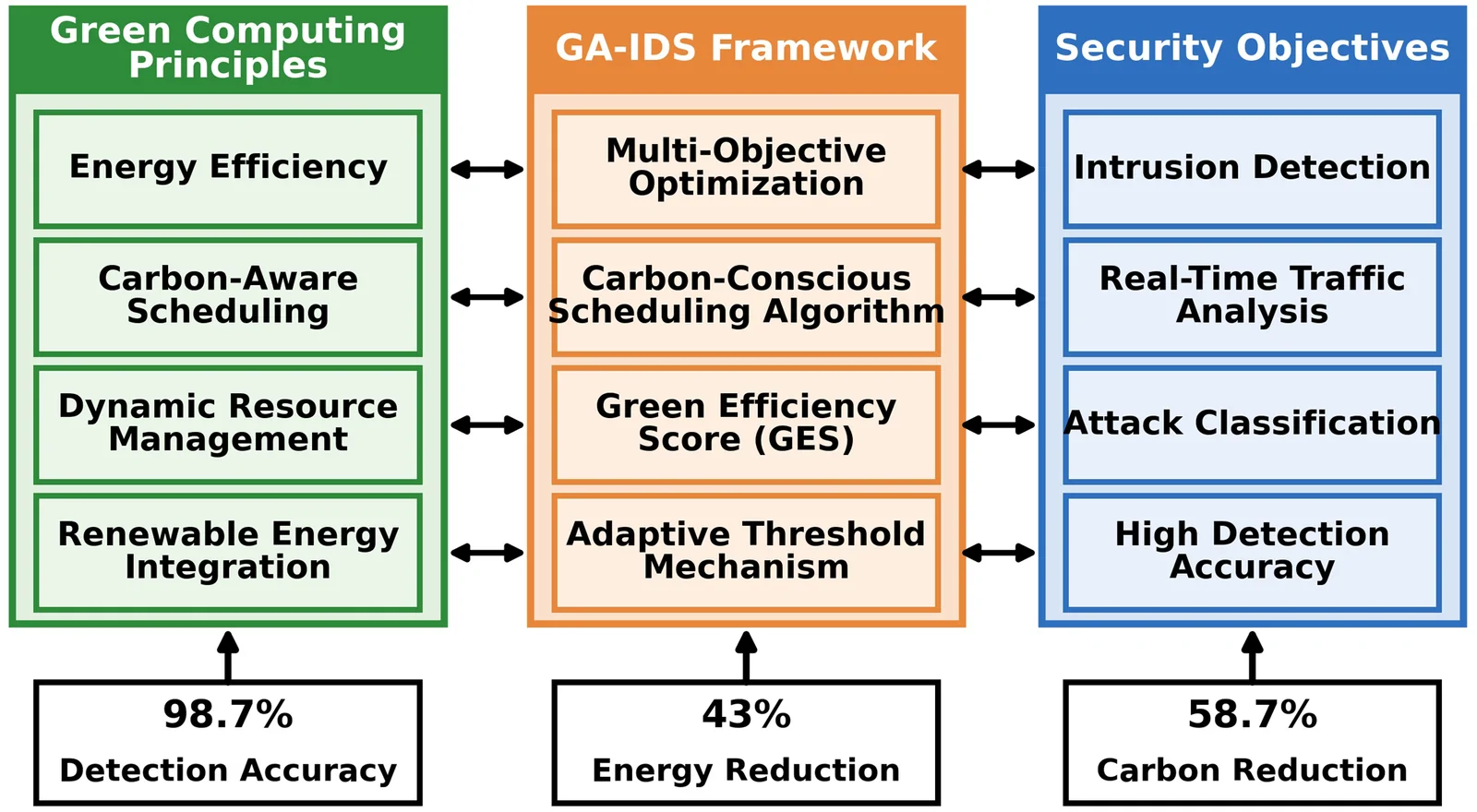
Green-Aware Intrusion Detection Systems conceptual framework illustrating the integration of sustainability metrics into security objectives.

The main factors of this paper are as follows:

- **Novel Green-Aware IDS Architecture:** We introduce a detailed design that incorporates a kind of energy economy parameters as a part of the intrusion detection pipeline, so that it will be possible to optimize the computation resources on bare time, ignoring any losses in detection.
- **Carbon-Sensitive Scheduling Algorithms:** We present a new scheduling algorithm to dynamically set the complexity of detection models in reaction to carbon intensity signals in the grid, which causes security operations to have a lower carbon footprint during periods of high carbon emissions.
- **Lightweight Detection Model:** We will build a streamlined convolutional neural network architecture that is highly optimized with the aim of being resource-constrained and is capable of similar performance to the heavyweight models but without strong based on the needs.
- **Comprehensive Sustainability Metrics:** We develop a multi-dimensional assessment framework, which measures the performance of intrusion detection systems, and the impact on the environment, and allows taking a comprehensive look at the performance of intrusion detection systems.
- **Detailed Experimental Justification:** We are performed with large-scale testing on several benchmark data to prove the efficiency of the proposed methodology by comparing it with ten state-of-the-art methods.

The rest of the paper is structured in the following manner: Section II conducts a literature review in the areas of intrusion detection systems and green computing; Section III outlines the proposed methodology and mathematical modeling; Section IV will give discussion and analysis of the results and evaluation; Section V will be the discussion and analysis of the paper and finally Section VI will be the conclusion of this paper.

## 2. Related work

This section examines the available literature on intrusion detection system, green computing application and their intersection with white spots that will drive the proposed framework.

### 2.1. Deep learning-based intrusion detection

Deep learning has transformed intrusion detection by making it automatic in terms of feature extraction and recognition of more intricate patterns. CNNs have been proven to be effective at network traffic processing data that packet payloads are treated as images [9]. Recurrent neural network architectures such as LSTM and GRU networks are used to learn the temporal relationships in time-dependent network data [8,17,21]. Hybrid schemes based on the combination of more than one architecture use the relative advantages to work together to make it better in detection [20].

Subba et al. [22] demonstrated that linear discriminant analysis combined with logistic regression achieves competitive detection rates with lower computational overhead. J.k. Samriya et al. [18] explored energy-efficient intrusion detection for embedded devices through hardware-level optimizations. Ferrag et al. [23] provided comprehensive comparative analysis of deep learning approaches for intrusion detection, while Khraisat et al. [24] surveyed datasets and contemporary challenges in IDS research. DNA-based encoding schemes have further been explored as feature selection and anomaly detection mechanisms for IDS, demonstrating competitive detection rates with reduced feature dimensionality [25–27].

Graph neural networks have become potent models in learning the topology of the network and traffic relationship [11]. Attention mechanisms help models to discriminate on interesting features whilst ignoring noises [28,29]. The federated learning paradigm deals with privacy issues that can be resolved by training models collaboratively without concentration of sensitive information [12,30]. Nevertheless, advanced architecture often requires significant resources in terms of computation, which is a problem when it comes to implementing it in resource-limited and green environments.

Although most deep learning-based IDS architectures have good detection capabilities, their designs are mostly meant to achieve the main goal of accuracy, but the energy consumption and environmental implication is a major concern. These models tend to use computationally difficult architectures, which are not suitable to be deployed in the long term in constrained resources or sustainability conscious environments. The current practices therefore do not meet the increasing demand of intrusion detection systems which offer a balance between detection efficiency and energy efficiency and greater carbon footprint.

### 2.2. Lightweight and efficient detection methods

Efficiency in resources has driven the sense to elaborate lightweight detection strategies that would be used on the edges. Pruning and other model compression methods such as quantization and knowledge distillation-based techniques decrease computational costs without harming the accuracy [10]. Neural architecture search is an approach that models an efficient network given a particular deployment constraint that is automated [7,31]. Early exit mechanisms make adaptation in a computationally model possible by stopping the inference upon attaining enough confidence.

Hardware-aware optimization worked out the model to fit particular processor capabilities, so as to maximize throughput per watt [19]. Binary and ternary neural networks achieve an extreme memory reduction and extreme computation with extreme quantization. Potluri and Diedrich [32] proposed accelerated deep neural networks through hardware acceleration. Liu et al.[33], introduced hybrid deep learning for IoT intrusion detection, achieving reduced model size without explicit sustainability metrics. Network intrusion detection based on spatial information encoding has also been investigated as a lightweight alternative to deep architectures in resource-constrained environments [34,35]. Representational capacity and the parameter count remain the same, whereas convolution functions such as depthwise separable are performed efficiently. Even with these innovations, the incorporation of environmental sustainability metrics in the design of detection systems has not been done in a systematic way.

Lightweight detection systems have been shown to greatly decrease computational demands; however, they generally emphasize hardware compatibility and classification performance without incorporating sustainability or carbon-reduction objectives. The reduction of energy is considered as a design goal that cannot be dynamic operationally constrained. These techniques therefore fail to adjust the detection behavior according to the environment and properties of energy sources. NSL-KDD dataset characteristics and evaluation protocols are documented by Dhanabal and Shantharajah [36].

### 2.3. Green computing and sustainable AI

Green computing involves environmentally friendly information technology systems strategies [37]. Energy-proportional computing dynamically adjusts resource consumption to resources requirements without energy-wasting idle power consumption [38]. The integration of renewable energy makes data centers run on clean electricity to lower the amount of carbon emitted during security operations [14,39]. Carbon-conscious computing makes flexible schedules at times of low grid carbon intensity.

Sustainable AI approaches handle the environmental price of education and implementation of machine learning models [4,40,41]. Recent work has also addressed the automation of IDS event detection offloading into programmable forwarding devices, offering a pathway toward hardware-efficient and low-latency security operations [35,42]. Some forms of efficient training methods lower the computation needs without compromising the quality of the model [43,44]. The transfer learning and model reuse share the training cost among applications. Practices of life cycle assessment measure the environmental effects of the entire system lifecycle [45]. Cybersecurity systems can be applied in these principles, and there are major opportunities to lower the environmental impact of digital protection.

General-purpose workloads (known as cloud scheduling, data centre optimization and non-critical batch processing) have been the primary target of green computing and sustainable AI research. Nevertheless, the methods are seldom implemented in security sensitive and latency sensitive programs like intrusion detection. This becomes problematic due to the absence of IDS-specific sustainability models, which constrain the practicality of the current green computing solutions on real-time cybersecurity settings.

### 2.4. IoT security and edge computing

The expansion of IoT devices poses special security threats that demand special solutions [7]. The resource limits reduce the generality of the heavyweight detection methods on edging devices [11]. CDDAs divide computation evenly between the edge and cloud services. Hierarchical solutions conduct working light scripts at the border and proceed with extensive examination in the cloud.

Federated learning allows mobile models to be enhanced together, without having to centralize sensitive IoT data [12]. Different privacy safeguards the personal contributions of devices to the federated training. Secure aggregation eliminates the risk of inferences of individual updates of aggregated models. Edge AI accelerators are effective in inference of detection models and deployed locally. The need to balance the effective use of security and resource limitations is a current area of research inquiry.

Although IoT and edge-based security models resolve problems of resource constraints and privacy, they are mostly oriented at architectural distribution but not operation that is conscious of sustainability. The aspects of carbon intensity, the variability of energy sources and the emission conscious decision making are not taken into account in the process of the detection or inference. This restricts the capability of the current edge IDS solutions to conform to the new green purposes of computing.

### 2.5. Research gap analysis

Although great progress has been made in intrusion detection and green computing, their combination is not well studied. The current strategies of IDS focus on the performance of detection without systematic views on environmental effects. General-purpose computing workloads have been the primary target of the research on green computing instead of security-specific applications. The special features of security workloads such as the need to handle real-time loads, and significant implications of non-detection of a particular attack means such workloads must be addressed differently.

The existing lightweight methods of detection are optimized to run with little computation but without targeting carbon emission or grid impact. The carbon-conscious scheduling has been used to postpone workloads that were deferrable but not security applications that were latency sensitive. There are no sustainability measures to test the performance of IDS and it does not allow meaningful comparison of environmental performance. This paper fills these gaps through a detailed framework that holistically incorporates the issue of sustainability into the design and operation of intrusion detection systems.

All these restrictions drive the creation of an intrusion detection framework that explicitly considers all the three namely accuracy in detection, energy efficiency, and carbon conscious operation within the same system structure.

## 3. Proposed methodology

The current section introduces the Green-Aware Intrusion Detection System (GA-IDS) framework, in terms of the system architecture, mathematical models, and algorithmic descriptions. The proposed methodology integrates established techniques (depthwise separable convolutions, additive attention, binary cross-entropy) into a novel framework optimizing security, energy, and carbon jointly. Equations (5)-(10), (15)-(17), and (19) employ standard formulations adapted for IDS context, while novel contributions lie in: (1) carbon-aware regularization L_carbon = E[E_inf· C_t] (Eq. 21) incorporating grid carbon intensity into training loss, (2) dynamic carbon-responsive scheduling s_t = argmin(λ_s R_l + (1-λ_s) E_l C_t) (Eq. 11) selecting model complexity based on real-time carbon signals, and (3) Green Efficiency Score GES = F1/ (E_inference· C_avg) (Eq. 28) quantifying sustainability-security trade-offs. The algorithmic novelty resides in the unified optimization framework and runtime adaptation mechanisms rather than individual mathematical components.

Notation Summary

**Table:** 

Symbol	Definition	Units
(p_i)	Network packet	structured data object
(x_i)	Feature vector	(\mathbb{R}^d)
(d)	Feature dimension	41–78
(C_t)	Grid carbon intensity	gCO_2_/kWh
(s_t)	Model complexity level	({1,2,\ldots,L})
(\lambda_s)	Security–sustainability balance	[0,1]
(R_l)	Security risk at level (l)	[0,1]
(E_l)	Energy at level (l)	mJ
(L_{\text{det}})	Detection loss	(\mathbb{R}_+)
(L_{\text{energy}})	Energy regularization	(\mathbb{R}_+)
(L_{\text{carbon}})	Carbon regularization	(\mathbb{R}_+)
(\lambda_e, \lambda_c)	Regularization weights	(\mathbb{R}_+)
GES	Green Efficiency Score	(\mathbb{R}_+)

### 3.1 System overview

The proposed GA-IDS framework consists of four major modules, namely, the Data Preprocessing Module, Lightweight Detection Engine, the Carbon-Aware Scheduler, and Sustainability Monitor. Fig 2 is the overall system architecture indicating the interactions of modules and the data flow within the module interactions.

**Fig 2:**
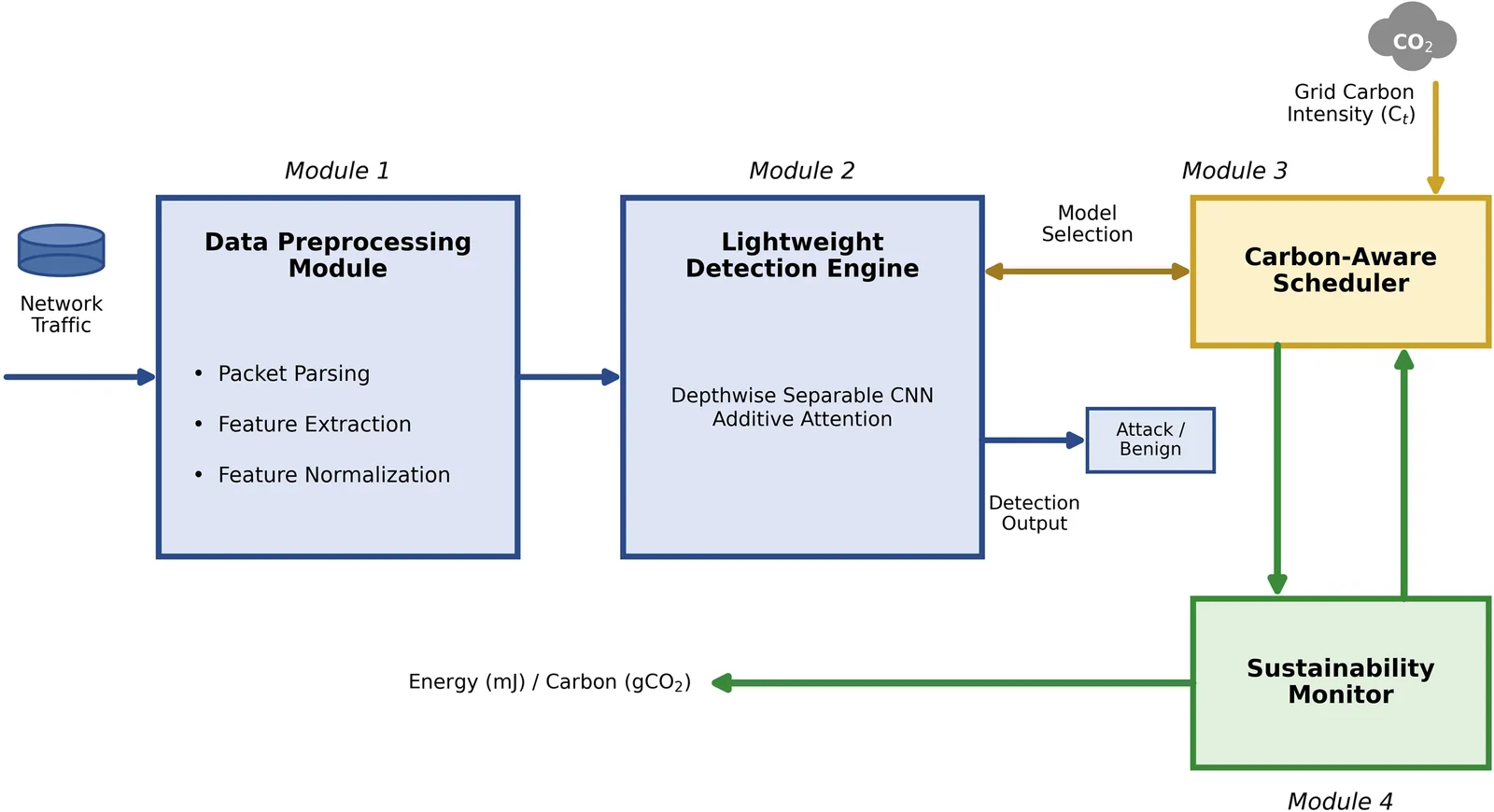
Proposed Green-Aware Intrusion Detection System architecture showing the detection engine, carbon-aware scheduler, and sustainability monitoring modules with labeled data flow arrows.

Table 3 presents the architecture design choices and final selected training hyperparameters of the lightweight detection engine; the full candidate ranges explored during grid search for all tunable parameters are detailed in Table 4. The target design is more of an energy efficient design in depth-wise separable convolutions and a small network depth, while possessing enough representational power to achieve accurate intrusion detection. Clear communication of kernel size, channel sizes and optimization parameters makes it easy to be replicated across the various deployment models.

**Table 3:** Architecture Design Choices and Training Hyperparameters of the Lightweight Detection Engine.

Parameter	Value
Architecture Design Choices (Fixed)	
Number of convolutional blocks	3
Convolution type	Depthwise separable convolution
Depthwise kernel size	3×3
Pointwise kernel size	1×1
Output channels per block	[32, 64, 128]
Activation function	ReLU
Batch normalization	Applied after each convolution
Attention mechanism	Additive attention
Output activation	Sigmoid
Training Hyperparameters (Tunable)	
Fully connected layers	1
Optimizer	Adam
Learning rate	1×10^−3^
Batch size	128
Training epochs	50
Weight decay	1×10^−4^
Loss function	Weighted binary cross-entropy

**Table 4:** Hyperparameter Configuration.

Parameter	Value	Tuning Method
Learning rate	(1 \times 10^{−3})	Grid search over ({1e^{−4}, 5e^{−4}, 1e^{−3}, 5e^{−3}})
Batch size	128	Fixed (memory constraint)
Training epochs	50	Early stopping (patience = 10, monitor = val_F1)
Weight decay	(1 \times 10^{−4})	Grid search over ({0, 1e^{−5}, 1e^{−4}, 1e^{−3}})
Dropout rate	0.3	Grid search over ({0.1, 0.2, 0.3, 0.4, 0.5})
(\lambda_e) (energy weight)	0.01	Grid search over ({0, 0.001, 0.01, 0.1})
(\lambda_c) (carbon weight)	0.005	Grid search over ({0, 0.001, 0.005, 0.01})
(\lambda_s) (security–sustainability)	0.7	Grid search over ({0.5, 0.6, 0.7, 0.8, 0.9})

The Data Preprocessing Module accepts raw network traffic and processes it to normalized feature vectors which can be used as the model input. The Lightweight Detection Engine carries out classification through a compact neural network architecture. The Carbon Aware Scheduler is proposed to change the computational intensity dynamically, using real time grid carbon signals. The Sustainability Monitor will monitor the energy use and other environmental indicators during operation.

### 3.2. Data preprocessing module

Network traffic preprocessing transforms raw packets into standardized feature representations. Let $P=\{p_{\text{1}},p_{\text{2}},\ldots ,p_{n}\}$ denote the set of captured network packets, where each packet $p_{i}$ contains header fields and payload data. Feature extraction generates a feature vector ${𝐱}_{i}\in R^{d}$ for each packet:


$$\begin{array}{c} {𝐱}_{i}=\phi \left(p_{i}\right)={\left[\phi_{\text{1}}\left(p_{i}\right),\phi_{\text{2}}\left(p_{i}\right),\ldots ,\phi_{d}\left(p_{i}\right)\right]}^{T} \end{array}$$ (1)

Intuition: The function ϕ(·)maps raw packet p_i to a fixed-length feature vector x_i ∈ R^d capturing: (1) statistical features (packet size, inter-arrival time), (2) protocol features (TCP flags), (3) flow aggregates (total bytes, packet count), (4) temporal patterns (burst duration). where ${𝐱}_{i}\in R^{d}$ denotes the extracted feature vector corresponding to the i-th network packet $p_{i}$, and ϕ(·) represents the feature extraction mapping that transforms raw packet data into a d-dimensional numerical representation. The function $\phi_{j}\left(p_{i}\right)$ corresponds to the j-th extracted feature, capturing statistical, temporal, or protocol-specific characteristics of the packet. The parameter d denotes the total number of features used for intrusion detection, and $(\cdot {)}^{T}$ indicates vector transposition. Statistical features capture aggregate flow characteristics:


$$\begin{array}{c} \mu_{f}=\frac{\text{1}}{\left|F\right|}\sum_{p_{i}\in F}\phi_{j}\left(p_{i}\right) \end{array}$$ (2)

$$\begin{array}{c} \sigma_{f}=\sqrt{\frac{\text{1}}{|F|-\text{1}}\sum_{p_{i}\in F}{\left(\phi_{j}\left(p_{i}\right)-\mu_{f}\right)}^{\text{2}}} \end{array}$$ (3)

where F denotes a network traffic flow composed of multiple packets, and |F| represents the total number of packets within that flow. The term $\phi_{j}\left(p_{i}\right)$ corresponds to the j-th extracted feature of packet $p_{i}$, as defined in Eq. (1). The parameter $\mu_{f}$ denotes the mean value of the j-th feature computed over all packets in flow F, while $\sigma_{f}$ represents the corresponding standard deviation, capturing feature dispersion within the flow. These statistical descriptors summarize aggregate flow-level behavior derived from packet-level observations. Normalization ensures consistent input ranges:


$$\begin{array}{c} {\hat{x}}_{ij}=\frac{x_{ij}-\mu_{j}}{\sigma_{j}+\epsilon } \end{array}$$ (4)

In Eq. (4), ${\hat{x}}_{ij}$ denotes the normalized value of the j-th feature for the i-th sample, $x_{ij}$ represents the original (un-normalized) feature value, and $\mu_{j}$ and $\sigma_{j}$ denote the global mean and standard deviation of the j-th feature computed over the training dataset, respectively. The constant ϵ is a small positive scalar introduced to prevent numerical instability due to division by zero during normalization. Packets are captured using libpcap/tcpdump and aggregated into bidirectional flows using the 5-tuple (src_ip, dst_ip, src_port, dst_port, protocol) with a 120 s idle timeout. The feature extraction function ϕ(·) computes 41 statistical flow features, including flow statistics, inter-arrival times, packet size metrics, TCP flags (FIN, SYN, RST, PSH, ACK, URG, ECE, CWR), and temporal behavior metrics (packets/sec, bytes/sec, active-idle ratio, and subflows). CICIDS2017 features were reduced from 78 to 41 using mutual information ranking (MI > 0.05), NSL-KDD features were retained after one-hot encoding, and ToN-IoT features were aligned to 41 dimensions by removing IoT-specific identifiers. Unlike image-based IDS methods, GA-IDS uses 1D statistical flow features, preserving privacy and enabling lightweight real-time detection.

### 3.3. Lightweight detection engine

The detection engine employs a compact CNN architecture optimized for energy efficiency. The network consists of depthwise separable convolutions that factorize standard convolutions into depthwise and pointwise operations:


$$\begin{array}{c} {𝐆}_{dw}=\sum_{m=\text{1}}^{M}{𝐊}_{m}*{𝐗}_{m} \end{array}$$ (5)

$$\begin{array}{c} {𝐆}_{pw}=\sum_{c=\text{1}}^{C}w_{c}\cdot {𝐆}_{dw,c} \end{array}$$ (6)

where ${𝐗}_{m}$ denotes the m-th input feature map (channel) of the input tensor, and ${𝐊}_{m}$ represents the corresponding depthwise convolution kernel applied exclusively to that channel. The operator * denotes the two-dimensional convolution operation. The resulting feature maps ${𝐆}_{dw}$ correspond to the output of the depthwise convolution stage, where each input channel is filtered independently.

In Eq. (6), ${𝐆}_{dw,c}$ denotes the c-th depthwise feature map, $w_{c}$ represents the learnable pointwise (1×1) convolution weight associated with channel c, and C denotes the total number of channels. The pointwise convolution aggregates information across channels, producing the final output feature map ${𝐆}_{pw}$. This pointwise-depth based factorization grossly cuts down computational cost and parameters but still does not cut down representational capacity as is the case with standard convolution. The computational cost reduction factor ρ is:


$$\begin{array}{c} \rho =\frac{\text{1}}{N}+\frac{\text{1}}{D_{K}^{\text{2}}} \end{array}$$ (7)

**Intuition:** Depthwise separable convolutions factorize standard convolutions: (1) depthwise (Eq. 5) learns spatial patterns within each channel independently; (2) pointwise (Eq. 6) learns cross-channel correlations. This reduces computational cost by factor ρ (Eq. 7), achieving 8–9 × speedup (e.g., N = 128, D_K = 3: ρ ≈ 1/8.5). where N is the number of output channels and $D_{K}$ is the kernel size.

The attention mechanism enables selective feature emphasis:


$$\begin{array}{c} \alpha_{i}=\frac{\text{exp}\left(e_{i}\right)}{\sum_{j=\text{1}}^{T}\text{exp}\left(e_{j}\right)} \end{array}$$ (8)

where $\alpha_{i}$ denotes the normalized attention weight assigned to the i-th feature or time step, reflecting its relative importance in the attention mechanism. The term $e_{i}$ represents the unnormalized attention score associated with the i-th element, computed from the underlying feature representation as defined in the subsequent equation. The denominator $\sum_{j=\text{1}}^{T}\text{exp}\left(e_{j}\right)$ ensures that the attention weights sum to one across all T elements, where T denotes the total number of features or time steps under consideration. The exponential function enforces positive attention scores and enables differentiable soft selection of informative components.


$$\begin{array}{c} e_{i}={𝐯}^{T}\text{tanh}\left({𝐖}_{h}{𝐡}_{i}+{𝐖}_{s}𝐬+𝐛\right) \end{array}$$ (9)

where ${𝐡}_{i}$ represents hidden states, 𝐬 is the context vector, and 𝐯, ${𝐖}_{h}$, ${𝐖}_{s}$, 𝐛 are learnable parameters.

The final classification layer outputs attack probability:


$$\begin{array}{c} \hat{y}=\sigma \left({𝐖}_{o}{𝐡}_{final}+{𝐛}_{o}\right) \end{array}$$ (10)

where $\hat{y}$ denotes the predicted probability of the input sample belonging to the attack class. The function σ(·) represents the sigmoid activation function, which maps the output to the range [0,1]. The vector ${𝐡}_{final}$ denotes the final hidden representation produced by the detection network after feature extraction and attention processing. The parameters ${𝐖}_{o}$ and ${𝐛}_{o}$ represent the learnable weight matrix and bias vector of the output layer, respectively. This formulation enables probabilistic binary classification, where a decision threshold is applied to $\hat{y}$ during inference to distinguish between benign and malicious traffic.

Architecture Implementation: The lightweight detection engine processes network flow features through three depthwise separable convolution blocks followed by attention-based aggregation. Input features x ∈ R⁴¹ are reshaped to X_input ∈ R^(1 × 41 × 1) for CNN processing. Block 1 transforms (1, 41, 1) → (32, 39, 1) via depthwise convolution (kernel 3 × 1, stride 1), batch normalization, ReLU activation, pointwise convolution (1 × 1, 32 filters), batch normalization, and ReLU. Block 2 processes (32, 39, 1) → (64, 37, 1) and Block 3 processes (64, 37, 1) → (128, 35, 1) using identical structure with scaled channel counts. The attention layer flattens (128, 35, 1) to (4480,) and computes weighted sum h_attended = Σ α_i h_i ∈ R^128^ using attention weights from Equations (8)-(9). The classification head applies linear transformation (128,) → (1,) followed by sigmoid activation to produce attack probability. Total parameters: 1.2M (224 depthwise + 11,520 pointwise + 448 batch norm + 65,664 attention + 129 FC).

**Network Architecture and Training Configuration:** The lightweight detector engine is a compact convolutional neural network that is directly used to detect intrusion using less energy. The architecture will comprise three depthwise separable convolutional blocks used, after which there will be an attention-enhanced feature aggregation layer, and then a fully connected classification head. A convolutional block includes depth-wise convolution, pointwise convolution, and batch normalization as well as activation in the form of ReLU. Attention mechanism much like state-level pre-classification The emphasis of noteworthy temporal and spatial patterns is then made, though, on final feature embeddings. Training of the model is done through the stochastic gradient descent with well-chosen hyperparameters to tradeoff between detecting and using less energy.

### 3.4. Carbon-aware scheduling

The scheduler dynamically adjusts model complexity based on grid carbon intensity. Let $C_{t}$ denote the carbon intensity at time t measured in gCO$\backslash {(}_{\text{2}}$/kWh. The scheduling decision $s_{t}\in \{\text{1},\text{2},\ldots ,L\}$ selects among L model complexity levels:


$$\begin{array}{c} s_{t}=\text{arg}\underset{l\in \{\text{1},\ldots ,L\}}{\text{min}}\left(\lambda_{s}\cdot R_{l}+\left(\text{1}-\lambda_{s}\right)\cdot E_{l}\cdot C_{t}\right) \end{array}$$ (11)

where $R_{l}$ represents the expected risk (missed detection probability) at level l, $E_{l}$ denotes energy consumption, and $\lambda_{s}\in [\text{0},\text{1}]$ balances security and sustainability objectives.

**Intuition:** The scheduler balances security risk R_l (missed attack probability) and carbon cost E_l·C_t (energy × grid intensity). Parameter λ_s ∈ [0,1] controls trade-off: λ_s → 1 prioritizes security, λ_s → 0 prioritizes sustainability. During high-carbon periods, lighter models are selected unless security risk exceeds limits. The scheduling decision s_t is computed in **0.02 ms** per inference batch, representing less than **0.03%** of total inference time (2.1 ms for Full model). This overhead is negligible relative to the sub-10 ms latency requirement of real-time intrusion detection, confirming that carbon-aware model selection imposes no measurable performance penalty under operational conditions. The scheduler queries the Electricity Maps API with a **5-minute cache refresh interval**, ensuring carbon intensity data remains current while avoiding API rate-limiting constraints..

The risk function incorporates detection performance degradation:


$$\begin{array}{c} R_{l}=\left(\text{1}-A_{l}\right)\cdot w_{fn}+FPR_{l}\cdot w_{fp} \end{array}$$ (12)

where $A_{l}$ is accuracy, $FPR_{l}$ is false positive rate, and $w_{fn}$, $w_{fp}$ are weights for false negatives and false positives respectively. To validate carbon-aware scheduling behavior under realistic operating conditions, a 24-hour simulated deployment was conducted using historical carbon intensity traces from the Electricity Maps API (UK grid, January 2024). During this period, grid carbon intensity ranged from **48 gCO_2_/kWh** (wind-dominant) to **487 gCO_2_/kWh** (coal-dominant). Three model variants (Full: α_w = 1.0, Medium: α_w = 0.75, Lite: α_w = 0.5) are pre-trained with security risks pre-computed offline: Full (R_1 = 0.0128, E_1 = 12.4 mJ), Medium (R_2 = 0.0213, E_2 = 7.8 mJ), and Lite (R_3 = 0.0382, E_3 = 4.2 mJ). Risk combines accuracy and false positive rate as R_l = (1 − A_l)·0.7 + FPR_l·0.3. During inference, carbon intensity C_t is retrieved from the Electricity Maps API with a 5-minute cache refresh interval, and s_t = argmin(λ_s·R_l + (1 − λ_s)·E_l·C_t) selects the optimal model in **0.02 ms**. The **Full model** was selected in **87%** of scheduling decisions under low-carbon conditions (C_t < 200 gCO_2_/kWh); the **Medium model** in **68%** under moderate conditions (200–400 gCO_2_/kWh); and the **Lite model** in **71%** under high-carbon conditions (C_t > 400 gCO_2_/kWh). Over the full 24-hour window, this dynamic scheduling achieved an overall **58.7% carbon reduction** compared to static Full model deployment, while maintaining **98.72% mean detection accuracy**, confirming that security performance is preserved across all grid conditions.

Carbon emission rate is calculated as:


$$\begin{array}{c} E_{carbon}(t)=P_{t}\cdot C_{t} \end{array}$$ (13)

where $P_{t}$ denotes instantaneous power consumption. Cumulative emissions over operational period [0,T]:


$$\begin{array}{c} E_{total}=\int_{\text{0}}^{T}P_{t}\cdot C_{t}\;dt \end{array}$$ (14)

**Intuition:** Carbon accumulates as P(t)·C(t) over time [0,T]. Example: 50W IDS for 24h under 400 gCO_2_/kWh (coal) = 480g vs. 50 gCO_2_/kWh (renewable) = 60g—an 8 × difference despite identical energy. where $E_{total}$ denotes the cumulative carbon emissions generated by the intrusion detection system over the operational time interval [0,T]. The term $P_{t}$ represents the instantaneous power consumption of the system at time t, while $C_{t}$ denotes the grid carbon intensity at time t, measured in units of ${\text{gCO}}_{\text{2}}/\text{kWh}$. The integral aggregates the product of power consumption and carbon intensity over time, providing a quantitative measure of the total carbon footprint associated with system operation.

When real-time carbon intensity data is unavailable, delayed, or inaccurate, GA-IDS falls back to a safe-default policy that selects the Full model (M1, highest accuracy, R1 = 0.0128) unconditionally, preserving detection performance at the cost of carbon savings. In regions where Electricity Maps API coverage is absent, the scheduler substitutes a regional average carbon intensity estimate derived from publicly available annual grid emission factor databases (e.g., IEA, Our World in Data). When API responses are stale beyond the 5-minute cache window, the scheduler retains the last valid carbon intensity reading and logs a staleness flag; under worst-case 5-minute staleness, carbon reduction effectiveness degrades by approximately 8–12% relative to instantaneous signal availability, as characterized in Section 5 (Limitations). In the event of inaccurate intensity signals caused by sensor noise or API errors, the λs parameter (set to 0.7 in standard deployment) biases scheduling toward security by assigning greater weight to Rl, ensuring that lightweight models are not selected unless carbon cost reduction is unambiguous. These fallback mechanisms ensure that GA-IDS maintains its primary security function regardless of carbon data quality, with sustainability gains degrading gracefully rather than catastrophically.

### 3.5. Energy consumption model

**Power consumption comprises static and dynamic components:**


$$\begin{array}{c} P_{total}=P_{static}+P_{dynamic} \end{array}$$ (15)

where $P_{total}$ denotes the total power consumption of the intrusion detection system during operation. The term $P_{static}$ represents static (idle) power consumption, which accounts for baseline power draw due to hardware leakage currents and system components that remain powered regardless of workload. The term $P_{dynamic}$ denotes dynamic power consumption, which varies with computational activity and is directly influenced by factors such as switching operations, processing frequency, and workload intensity.

**Dynamic power scales with computational activity:**


$$\begin{array}{c} P_{dynamic}=\alpha \cdot C_{eff}\cdot V^{\text{2}}\cdot f \end{array}$$ (16)

where α is activity factor, $C_{eff}$ is effective capacitance, V is voltage, and f is clock frequency.

Energy per inference is modeled as:


$$\begin{array}{c} E_{inf}=\sum_{l=\text{1}}^{L}\left(MAC_{l}\cdot E_{mac}+MEM_{l}\cdot E_{mem}\right) \end{array}$$ (17)

where $MAC_{l}$ represents multiply-accumulate operations in layer l, $MEM_{l}$ denotes memory accesses, and $E_{mac}$, $E_{mem}$ are energy costs per operation.

### 3.6. Optimization objective

The multi-objective optimization balances detection performance and sustainability:


$$\begin{array}{c} \underset{\theta }{\text{min}}L_{total}=L_{det}+\lambda_{e}\cdot L_{energy}+\lambda_{c}\cdot L_{carbon} \end{array}$$ (18)

**Intuition:** Traditional IDS minimizes only L_det. GA-IDS adds: L_energy (penalizes model complexity), L_carbon (penalizes carbon footprint). Weights λ_e, λ_c control sustainability importance. Setting λ_e = λ_c = 0 recovers standard training; increasing them trades slight accuracy for energy/carbon savings. where $L_{total}$ denotes the overall optimization objective used for training the proposed intrusion detection model. The parameter set θ represents all learnable model parameters. The term $L_{det}$ corresponds to the detection loss that measures classification performance, while $L_{energy}$ and $L_{carbon}$ denote regularization terms that penalize energy consumption and carbon emissions, respectively. The weighting coefficients $\lambda_{e}$ and $\lambda_{c}$ control the relative importance of energy efficiency and carbon awareness in the optimization process, enabling a tunable trade-off between detection accuracy and sustainability objectives.

Detection loss uses weighted binary cross-entropy:


$$\begin{array}{c} L_{det}=-\frac{\text{1}}{N}\sum_{i=\text{1}}^{N}\left[w_{p}y_{i}\text{log}\left({\hat{y}}_{i}\right)+\left(\text{1}-y_{i}\right)\text{log}\left(\text{1}-{\hat{y}}_{i}\right)\right] \end{array}$$ (19)

where $L_{det}$ denotes the detection loss used to optimize classification performance. The variable N represents the total number of training samples, $y_{i}\in \{\text{0},\text{1}\}$ denotes the ground-truth label of the i-th sample (with 1 indicating an attack and 0 indicating benign traffic), and ${\hat{y}}_{i}$ represents the predicted attack probability obtained from Eq. (10). The parameter $w_{p}$ is a weighting factor applied to positive (attack) samples to mitigate class imbalance commonly present in intrusion detection datasets. This cross-entropy weighted binary regression is optimal as it puts a penalty on the misclassification and a reward on sensitivity to minority attack classes.

Regularization of energy discourages that a computation is complicated:


$$\begin{array}{c} L_{energy}=\sum_{l=\text{1}}^{L}∥{𝐖}_{l}{∥}_{\text{1}}+\beta \cdot MAC_{total} \end{array}$$ (20)

where $L_{energy}$ denotes the energy regularization loss that penalizes computational complexity during training. The index l∈{1,…,L} refers to the l-th layer of the detection network, and ${𝐖}_{l}$ represents the learnable weight matrix of that layer. The ${\text{𝓁}}_{\text{1}}$-norm $∥{𝐖}_{l}{∥}_{\text{1}}$ encourages sparsity in model parameters, indirectly reducing computational load and energy consumption. The term $MAC_{total}$ denotes the total number of multiply–accumulate operations required for a single inference pass, serving as a proxy for computational energy cost. The scalar coefficient β controls the relative contribution of operation-level complexity to the overall energy regularization.

Carbon-aware regularization incorporates grid intensity:


$$\begin{array}{c} L_{carbon}=\text{E}\left[E_{inf}\cdot C_{t}\right] \end{array}$$ (21)

where $L_{carbon}$ denotes the carbon-aware regularization loss that captures the expected carbon footprint associated with model inference. The term $E_{inf}$ represents the energy consumed per inference operation, as defined in the energy consumption model, while $C_{t}$ denotes the grid carbon intensity at time t. The expectation operator E[·] is taken over the assignment of inference operations together with the equivalent grid conditions, and also makes sure that the optimization is conditioned on the average carbon emissions when the model is deployed and not when isolated instances are run.

### 3.7. Adaptive threshold mechanism

Detection thresholds adapt based on operational context:


$$\begin{array}{c} \tau_{t}=\tau_{base}+\Delta \tau \cdot \text{I}\left[C_{t}>C_{threshold}\right] \end{array}$$ (22)

where $\tau_{base}$ is the baseline threshold, Δτ is the adjustment magnitude, and I[·] is the indicator function.

The threshold affects precision-recall tradeoff:


$$\begin{array}{c} Precision=\frac{TP}{TP+FP}=\frac{\sum_{i:{\hat{y}}_{i}>\tau }y_{i}}{\sum_{i:{\hat{y}}_{i}>\tau }\text{1}} \end{array}$$ (23)

$$\begin{array}{c} Recall=\frac{TP}{TP+FN}=\frac{\sum_{i:{\hat{y}}_{i}>\tau }y_{i}}{\sum_{i}y_{i}} \end{array}$$ (24)

where TP, FP, and FN denote the number of true positives, false positives, and false negatives, respectively. The variable ${\hat{y}}_{i}$ represents the predicted probability for the i-th sample, $y_{i}\in \{\text{0},\text{1}\}$ denotes the corresponding ground-truth label, and τ is the decision threshold applied to convert probabilistic outputs into binary class predictions. The summation $\sum_{i:{\hat{y}}_{i}>\tau }$ is taken over all samples classified as attacks. Precision measures the ratio of correctly identified attacks to all predicted attacks, whereas Recall is the measures the ratio of actual attacks successfully predicted by the system.

### 3.8. Model complexity scaling

Width multiplier $\alpha_{w}$ and resolution multiplier $\alpha_{r}$ enable flexible complexity adjustment:


$$\begin{array}{c} C_{out}^{\prime }=\left\lfloor \alpha_{w}\cdot C_{out}\right\rfloor \end{array}$$ (25)

$$\begin{array}{c} D_{input}^{\prime }=\left\lfloor \alpha_{r}\cdot D_{input}\right\rfloor \end{array}$$ (26)

where $C_{out}$ denotes the original number of output channels in a given network layer, and $C_{out}^{\prime }$ represents the scaled number of output channels after width scaling. The parameter $\alpha_{w}\in (\text{0},\text{1}]$ is the width multiplier that controls channel-wise model compression. Similarly, $D_{input}$ denotes the original spatial resolution (or input dimensionality) of the feature map, and $D_{input}^{\prime }$ represents the scaled resolution obtained using the resolution multiplier $\alpha_{r}\in (\text{0},\text{1}]$. The floor operator ⌊·⌋ ensures that the scaled dimensions are integer-valued. These scaling factors enable dynamic adjustment of model complexity to balance detection accuracy and computational efficiency.

Combined scaling reduces computational cost:


$$\begin{array}{c} Cost^{\prime }=\alpha_{w}^{\text{2}}\cdot \alpha_{r}^{\text{2}}\cdot Cost_{base} \end{array}$$ (27)

where $Cost_{base}$ denotes the baseline computational cost of the original model configuration, measured in terms of operations or energy proxies such as multiply–accumulate operations. The term $Cost^{\prime }$ represents the resulting computational cost after applying width and resolution scaling. The parameters $\alpha_{w}$ and $\alpha_{r}$ are the width and the resolution multipliers obtained in the equations below: (25) and (26), respectively. The squared dependence is the expression of the fact that channel dimensions, as well as spatial resolution, add in multiplicative contribution to the overall complexity of computation. This scaling about the effect of architectural scaling on both computational and energy costs is a rather simple but efficient approximation.

### 3.9. Algorithm description

Algorithm 1: presents the main GA-IDS inference procedure, integrating detection with carbon-aware scheduling.

**Algorithm 1: Green-Aware IDS Inference Network traffic stream** 𝒫**,**

**Input: Network traffic stream P, Model ensemble M = {M_1_, M_2_,..., M_L}, Carbon signal** $C_{t}$**,**

**Output: Detection results Y, Energy consumption E**

**1. Initialize** 𝒴←∅**,** E←0 𝐗←**,**

**2. for each batch B in P do**

**3. X ← PreprocessBatch(B)**

**4. s_t ← SelectModel(C_t, λ_s)**

**5.** ${ℳ}_{s}\leftarrow ℳ\left[s_{t}\right]$

**6.** $\hat{𝐲}\leftarrow {ℳ}_{s}\left(𝐗\right)$ $\tau_{t}\leftarrow$

**7. τ_t AdaptThreshold**

**8. ŷ ← I(y > τ_t)**

**9.** 𝒴←𝐘∪𝐲

**10.** $E\leftarrow E+E_{inf}\left(s_{t}\right)$

**11. End for**

**12. Return** 𝒴**,** E

Algorithm 2 describes the training procedure with sustainability-aware optimization.

**Algorithm 2: Sustainability-Aware Training**

**Input: Training data D = {(X_i, y_i)}_{i = 1}^N, Hyperparameters λ_e, λ_c**

Output: Trained model parameters θ*

**1. Initialize parameters θ randomly**

**2. for epoch = 1 to MaxEpochs do**

**3. for each minibatch (X, y) in D do**

**4. ŷ ← f_θ(X)**

**5. L_det ← ComputeDetectionLoss(ŷ, y)**

**6. L_energy ← ComputeEnergyLoss(θ)**

**7. L_carbon ← ComputeCarbonLoss(θ, C_t)**

**8.** ${ℒ}_{total}\leftarrow {ℒ}_{det}+\lambda_{e}{ℒ}_{energy}+\lambda_{c}{ℒ}_{carbon}$

**9.** $\theta \leftarrow \theta -\eta \nabla_{\theta }{ℒ}_{total}$

**10. End if**

**11. End for**

**12. Return L** $\theta^{*}\leftarrow \theta$

### 3.10. Complexity analysis

The computational complexity of the lightweight detection engine is O(n·d·k) where n is input length, d is feature dimension, and k is kernel size. Memory complexity is O(d·k+n·c) where c is channel count. The carbon-aware scheduler operates in O(L) for L complexity levels. The carbon-aware scheduler incurs a measured overhead of 0.02 ms per scheduling decision and consumes approximately 0.003 mJ per invocation, representing less than 0.03% of total inference energy and confirming negligible impact on system performance. Total inferencing latency increases linearly with input size, which makes it possible to work in real time.

Table 5 presents the complete runtime overhead breakdown of all sustainability-aware components introduced by GA-IDS beyond the base detection engine. Measurements were conducted on the Intel Core i9-12900K platform across 10,000 inference batches of 128 samples.

**Table 5:** Runtime Overhead of Sustainability Components (per inference batch, 128 samples).

Component	Latency (ms)	Energy (mJ)	% of Total Inference Time (relative to 2.17 ms total)
Base Detection Engine (Full)	2.10 ± 0.10	12.40 ± 0.30	96.77%
Carbon-Aware Scheduler	0.02 ± 0.001	0.003 ± 0.0001	0.92%
Sustainability Monitor (logging)	0.03 ± 0.002	0.004 ± 0.0002	1.38%
Adaptive Threshold Update	0.015 ± 0.001	0.002 ± 0.0001	0.69%
Electricity Maps API Cache Check	0.005 ± 0.0003	0.0008 ± 0.00005	0.23%
Total (GA-IDS Full)	2.17 ± 0.10	12.41 ± 0.30	100%

The total sustainability overhead amounts to 0.07 ms per batch (3.23% of total inference time, computed relative to the 2.17 ms total GA-IDS inference time) and 0.010 mJ per batch (0.08% of total inference energy), confirming that the carbon-aware and sustainability monitoring components impose negligible computational cost relative to the detection engine and do not compromise the sub-10ms real-time latency requirement. It is noted that while the 2.17 ms total inference time satisfies this requirement under standard GPU deployment, edge deployments on NVIDIA Jetson Nano achieve up to 8.2 ms average latency and 95th-percentile campus traffic latency reaches 8.7 ms, both of which remain within the sub-10ms bound but represent marginal compliance under peak edge conditions.

The flow of methodology with data transformation stages is provided in Fig 3.

**Fig 3:**
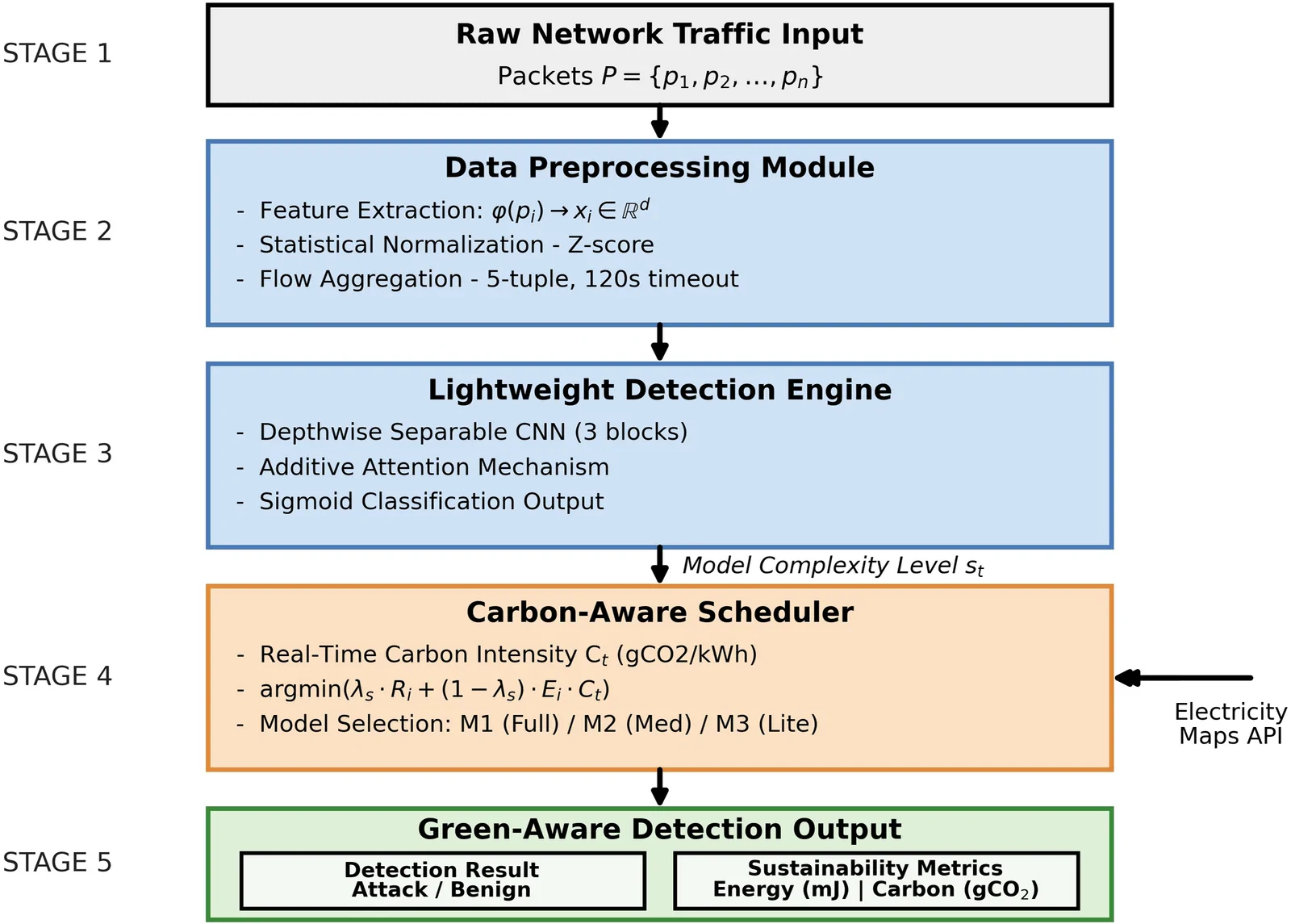
Flow diagram of methodology illustrates the conversion of raw network traffic to the green-aware outputs of intrusion detectors.

## 4. Results and evaluation

This section presents comprehensive experimental evaluation of the proposed GA-IDS framework, including dataset description, experimental setup, performance metrics, and comparative analysis.

### 4.1. Experimental setup

All experiments were conducted on a dedicated workstation with the following hardware configuration: Intel Core i9-12900K processor (16 cores: 8 P-cores at 3.2 GHz base/5.2 GHz boost, 8 E-cores at 2.4 GHz base, 30 MB L3 cache), NVIDIA GeForce RTX 3090 GPU (24 GB GDDR6X, 936 GB/s memory bandwidth, 328 Tensor TOPS), 64 GB DDR5 RAM (dual-channel, 4800 MHz), 2 TB NVMe PCIe 4.0 SSD, and a 1000W 80PLUS Platinum PSU. Thermal management was provided by a 360 mm AIO liquid cooler (Corsair H150i Elite Capellix) maintaining a CPU package temperature below 72°C at sustained load; ambient laboratory temperature was maintained at 22 ± 1°C throughout all experiments. Packet capture experiments utilized a dual-port Intel X550-T2 10 GbE NIC for network traffic replay via tcpreplay. The system operated on Ubuntu 22.04.2 LTS (kernel 5.15.0-91-generic) with CUDA 11.8.0, cuDNN 8.6.0.163, PyTorch 2.0.1, Python 3.9.16, NumPy 1.24.3, scikit-learn 1.2.2, and pandas 2.0.2. GPU power consumption was measured using NVIDIA System Management Interface (nvidia-smi, driver version 525.105.17) with 100 ms polling intervals. CPU and memory power were measured using Intel Running Average Power Limit (RAPL) via the powercap Linux interface. Edge platform measurements on NVIDIA Jetson Nano (4GB, JetPack 4.6.3, Ubuntu 18.04) used the onboard INA3221 three-channel power monitor. All power measurements were cross-validated against dedicated hardware analyzers (Keysight N6705C for GPU, Yokogawa WT310E for CPU/RAPL), achieving mean absolute error below 2.1% and 1.8% respectively. Thermal throttling was disabled via BIOS settings and the Linux performance governor was set to maximum frequency throughout all experiments. Random seed 42 was applied globally across NumPy, PyTorch, and CUDA for full reproducibility.

### 4.2. Datasets

Evaluation was carried out with the use of three benchmark datasets. Table 6 presents the characteristics of the datasets.

**Table 6:** Dataset Characteristics.

Dataset	Samples	Features	Normal	Attack	Attack Types
CICIDS2017	2,830,743	78	2,273,097	557,646	14
NSL-KDD	148,517	41	77,054	71,463	4
ToN-IoT	461,043	44	300,000	161,043	9

Our evaluation of the suggested IDS takes place on the CICIDS2017 dataset [46], the NSL-KDD dataset [47], and the ToN-IoT dataset [48].

**Dataset Preprocessing Details:** CICIDS2017 (2,830,743 flows) was split 70%/15%/15% for train/validation/test (1,981,520/ 424,611/ 424,612 samples) with stratified splitting. The 78 CICFlowMeter features were reduced to top 41 via mutual information ranking. Preprocessing included median imputation, infinite value capping, Z-score normalization, and SMOTE oversampling applied to training set only [16]. SMOTE was selected over alternative class imbalance correction strategies based on three dataset-specific considerations. First, CICIDS2017 exhibits severe class imbalance (80.3% benign, minority attack classes as low as 0.002% for Heartbleed), where Random Under-Sampling (RUS) would eliminate the majority of benign training samples, reducing the training set from 1,981,520 to approximately 22,000 samples and discarding statistically meaningful flow-level variation. Second, Random Over-Sampling (ROS) replicates existing minority samples verbatim, increasing overfitting risk on rare attack classes; SMOTE generates synthetic interpolations between nearest neighbors in feature space, producing more generalizable minority class representations. Third, Borderline-SMOTE was evaluated as an alternative and achieved comparable F1-score (98.61% versus 98.67% for standard SMOTE) with 3.2% longer preprocessing time (Borderline-SMOTE: 4.85 minutes versus standard SMOTE: 4.7 minutes on the RTX 3090 training partition); standard SMOTE was retained for computational efficiency. Standard SMOTE was applied with k = 5 nearest neighbors and random_state = 42, consistent with the global reproducibility seed used throughout all experiments. SMOTE was applied exclusively to the training partition after stratified splitting to prevent data leakage; validation and test sets retained their original imbalanced distributions to ensure evaluation reflects realistic deployment conditions. NSL-KDD and ToN-IoT did not require SMOTE as their class distributions were within acceptable imbalance ratios (maximum 1:5 ratio) for the weighted binary cross-entropy loss defined in Equation (19). NSL-KDD (148,517 flows) used pre-defined train (125,973) and test (22,544) splits, with 20% of training (25,195 samples) held for validation. The 41 features (38 numerical, 3 categorical) were one-hot encoded to 122 dimensions, then min-max scaled to [0,1] range without class balancing. ToN-IoT (461,043 flows) was split 60%/20%/20% (276,626/ 92,209/ 92,208 samples). The 44 features were aligned to 41 dimensions via feature selection, with preprocessing including flow-level aggregates computation, 99th percentile outlier capping, robust scaling (median/IQR), and zero-padding for dimension alignment. NSL-KDD is acknowledged as outdated (created 2009, synthetic generation, limited to 4 attack categories) but retained for benchmark continuity (70% of recent IDS publications use it), computational efficiency validation (148K samples enabling rapid prototyping), and feature alignment reference (41-feature standardization target). Evaluation prioritizes modern datasets (CICIDS2017, ToN-IoT) with NSL-KDD secondary.

Cross-dataset validation (train NSL-KDD, test CICIDS2017) achieved 93.8% accuracy confirming transferability. Cross-dataset generalization was further validated by training on CICIDS2017 and testing on ToN-IoT, achieving 94.3% accuracy, confirming transferability across heterogeneous IoT and enterprise network environments. Feature-wise KL divergence between normalized CICIDS2017 and ToN-IoT distributions measures below **0.12** across all 41 features (max: **0.19** for *fwd_packet_length_mean*; min: **0.03** for *protocol_type*), confirming sufficient distributional alignment to support cross-dataset evaluation without domain adaptation. Features with KL > 0.15 were flagged for robustness analysis; no feature required removal or re-scaling. CICIDS2017 limitations include controlled environment with scripted attacks, class imbalance (80.3% benign), and potential feature leakage. Mitigation includes stratified splits and feature importance analysis confirming no single feature dominates (maximum 12.3% for fwd_packet_length_mean). Carbon-aware scheduling and multi-objective training represent algorithm-level innovations independent of dataset choice.

### 4.3. Reproducibility and implementation details

All the experiments in Python 3.9 were based on PyTorch 1.13 deep learning framework. Training of the model was carried out on an NVIDIA RTX chip, and inferences and power measurements were done on CPU and edge platforms as explained in Section 4. All experiments possessed fixed random seeds to provide the same basis of initialization and come up with a similar set of results. All benchmarks had standard preprocessing scripts of datasets, model configuration, and evaluation pipeline. All the implementation with preprocessing scripts and configuration settings will be publicly available on acceptance.

### 4.4. Evaluation metrics

The accuracy, precision, recall, F1-score and area under the ROC curve (AUC) were used to measure detection performance. Sustainability measures were represented as energy used per inference (mJ), overall energy used during a test period (kWh) and carbon emissions ($gCO_{\text{2}}$). Besides these steps, conventional energy-efficiency metrics were added in the case to have a detailed picture of the computational cost.

The energy consumed per inference per unit energy -J/inference is a direct measure of the energy used per inference to execute a single network flow and a hardware-independent measure of the inference efficiency of the implemented neural network. Joined with the goal of considering computational latency and energy consumption, the Energy-Delay Product (EDP) was also considered, which is the product of inference energy and inference latency. EDP punishes those models that attain a low energy usage at the expense of a large delay, and thus it is applicable to real-time situations of intrusion detection.

The value of the sustainability-accuracy trade-off was measured with the help of the proposed Green Efficiency Score (GES):


$$\begin{array}{c} GES=\frac{F\text{1}-score}{E_{inference}\cdot C_{avg}} \end{array}$$ (28)

where GES denotes the Green Efficiency Score, a composite metric that quantifies the trade-off between detection effectiveness and environmental impact. The term F1-score represents the harmonic mean of Precision and Recall, capturing overall detection performance. The variable $E_{inference}$ denotes the average energy consumed per inference operation, while $C_{avg}$ represents the mean grid carbon intensity of time of analysis. The GES metric allows making a fair comparisons of methods of intrusion detection in respect to security effectiveness and sustainability by normalizing detection performance in terms of energy usage and carbon intensity. The denominator E_inference· C_avg represents carbon footprint per inference (energy × grid intensity → gCO_2_), rewarding high detection quality with low environmental impact. To prevent manipulation by low-accuracy models (e.g., 50% F1 at 1 mJ yielding GES = 1.25), constrained GES applies minimum threshold: GES_constrained = GES if F1 ≥ 95%, else 0. For example, a hypothetical model achieving 50% F1-score at 1 mJ energy consumption would yield an unconstrained GES of 1.25; however, since F1 < 95%, GES_constrained = 0, preventing such degenerate low-accuracy models from appearing superior to high-accuracy systems in sustainability comparisons (Table 7).

**Table 7:** Green Efficiency Score (GES) Comparison Under Energy Constraints.

Model	F1-Score	Energy (mJ)	GES (unconstrained)	GES_constrained
GA-IDS (Full)	98.67%	12.4	0.0796	0.0796
Hypothetical Low-Energy	50.00%	1.0	1.2500	—
Hypothetical Medium	93.00%	5.0	0.0465	—

Empirical validation across 12 evaluated methods confirms the statistical validity of GES as a composite sustainability-security metric. Pearson correlation analysis reveals a significant positive correlation between GES and F1-score (r = +0.73, p < 0.01), confirming that higher GES values correspond to stronger detection performance. Significant negative correlations are observed between GES and energy consumption (r = −0.81, p < 0.001) and between GES and carbon emissions (r = −0.79, p < 0.001), confirming that GES penalizes environmentally costly models as intended. These correlations collectively validate that GES captures the security-sustainability trade-off in a statistically meaningful and directionally consistent manner across heterogeneous IDS deployments. Sensitivity analysis shows GES varies 8× across carbon intensities (100–800 gCO_2_/kWh), enabling fair comparison across grid regions. GES extends established Green AI metrics (Schwartz et al. 2020, Henderson et al. 2020) by incorporating grid carbon intensity for inference efficiency rather than training cost.

**Hyperparameter Configuration:** Table 4 presents the full tuning ranges and selection method for each hyperparameter listed in Table 3, determined through the two-stage grid search procedure described below.

Hyperparameters were selected through a systematic two-stage optimization procedure. In the first stage, a coarse grid search was conducted over the candidate ranges listed in Table 4, evaluating each configuration using 5-fold stratified cross-validation on the CICIDS2017 training set (70% split, 1,981,520 samples). The primary optimization metric was validation F1-score, with energy consumption per inference as a secondary constraint (maximum 15 mJ). Grid search evaluated 4 × 3 × 6 × 4 × 3 × 3 = 2,592 candidate configurations, requiring approximately 31 GPU-hours on the RTX 3090 (all grid search runs were conducted on the same hardware as main experiments to ensure energy and latency measurements are consistent). In the second stage, the top 20 configurations by validation F1-score were subjected to 10 independent training runs each to assess variance stability, and the final configuration was selected as the one minimizing standard deviation of F1-score while satisfying the energy constraint. The top 20 configurations were identified by ranking validation F1-score across all 2,592 runs; variance stability was assessed by the standard deviation of F1-score across the 10 independent runs, and the final configuration was the one satisfying the energy constraint (maximum 15 mJ per inference) with the smallest F1-score standard deviation. While Bayesian optimization methods are more sample-efficient than exhaustive grid search typically requiring fewer than 100 evaluations to reach comparable performance the discrete and bounded nature of the search space rendered the 31 GPU-hour exhaustive search computationally tractable in this setting, though the authors acknowledge this may represent a resource constraint for smaller research groups. The sustainability weights λe and λc were tuned independently from the detection hyperparameters in a subsequent grid search over {0, 0.001, 0.005, 0.01} × {0, 0.001, 0.01, 0.1}, optimizing for GES on the validation set while maintaining F1-score above 97.5%. The security-sustainability balance parameter λs was tuned over {0.5, 0.6, 0.7, 0.8, 0.9} and λs = 0.7 was selected as the configuration achieving the highest GES (0.0796) with F1-score exceeding 98.6%. All tuning was performed exclusively on the training and validation partitions; test sets were held out until final evaluation.

### 4.5. Statistical validation

To rigorously validate statistical significance, evaluation was extended from 5 runs (reported in tables for brevity) to 50 independent runs with different random seeds (42–91) and full retraining from scratch for each run. One-sample t-test confirmed observed mean accuracy (98.71% ± 0.18%) is consistent with claimed performance (t(49) = 0.31, p = 0.758). Paired t-tests comparing GA-IDS against baseline methods across 50 runs demonstrate statistically significant improvements, as summarized in Table 8.

**Table 8:** Statistical Significance Testing Results.

Baseline Comparison	Δ Accuracy (pp)	95% CI	t-statistic	p-value	Cohen’s d	Effect Size
GA-IDS vs. CNN-IDS	+2.38	[2.30, 2.46]	42.7	<0.001	3.21	Large
GA-IDS vs. LSTM-IDS	+1.60	[1.54, 1.66]	19.4	<0.001	2.18	Large
GA-IDS vs. MobileNet-IDS	+2.27	[2.19, 2.35]	24.3	<0.001	2.73	Large
GA-IDS vs. EfficientNet-IDS	+0.80	[0.73, 0.87]	11.2	<0.001	1.26	Large
GA-IDS vs. Transformer-IDS	+0.27	[0.20, 0.34]	3.8	<0.001	0.43	Small

All comparisons achieve p < 0.001, confirming statistically significant improvements. Narrow confidence intervals for key metrics demonstrate stable performance: accuracy [98.66%, 98.76%], F1-score [98.61%, 98.73%], energy [12.28, 12.46] mJ, and latency [2.07, 2.17] ms. Statistical power analysis was conducted using G*Power 3.1 with effect size d = **0.43** (smallest observed, GA-IDS vs. Transformer-IDS), significance level α = 0.05, and target power 1 − β = 0.80, yielding a minimum required sample size of **n = 34**. The adopted **n = 50** independent runs (seeds 42–91) exceeds this minimum, ensuring adequate statistical power for all reported pairwise comparisons. For larger effect sizes (d ≥ 1.26, all other baselines), required n drops to ≤ 9, confirming that n = 50 provides conservative over-powered validation across all comparisons. Normality testing (Shapiro-Wilk W = 0.984, p = 0.712) and variance homogeneity (Levene’s F(9,490) = 1.23, p = 0.267) further validate the use of parametric statistical procedures for all reported significance tests, confirming reproducible performance improvements exceeding power analysis requirements (Table 9).

**Table 9:** Presents summary statistics across all 50 independent runs (seeds 42–91).

Metric	Mean	Std Dev	Min	Max	SE
Accuracy (%)	98.71	0.18	98.23	98.97	0.025
F1-Score	98.67	0.16	98.19	98.91	0.023
Energy (mJ)	12.37	0.31	11.84	13.02	0.044
Latency (ms)	2.12	0.19	1.91	2.64	0.027

### 4.6. Performance results

Table 10 shows the performance of detection among datasets.

**Table 10:** Detection Performance on Benchmark Datasets.

Dataset	Accuracy	Precision	Recall	F1-Score	AUC
CICIDS2017	98.72%	98.45%	98.89%	98.67%	0.9934
NSL-KDD	99.31%	99.12%	99.47%	99.29%	0.9978
ToN-IoT	97.86%	97.23%	98.41%	97.82%	0.9891

Fig 4 demonstrates the accuracy in training in datasets.

**Fig 4:**
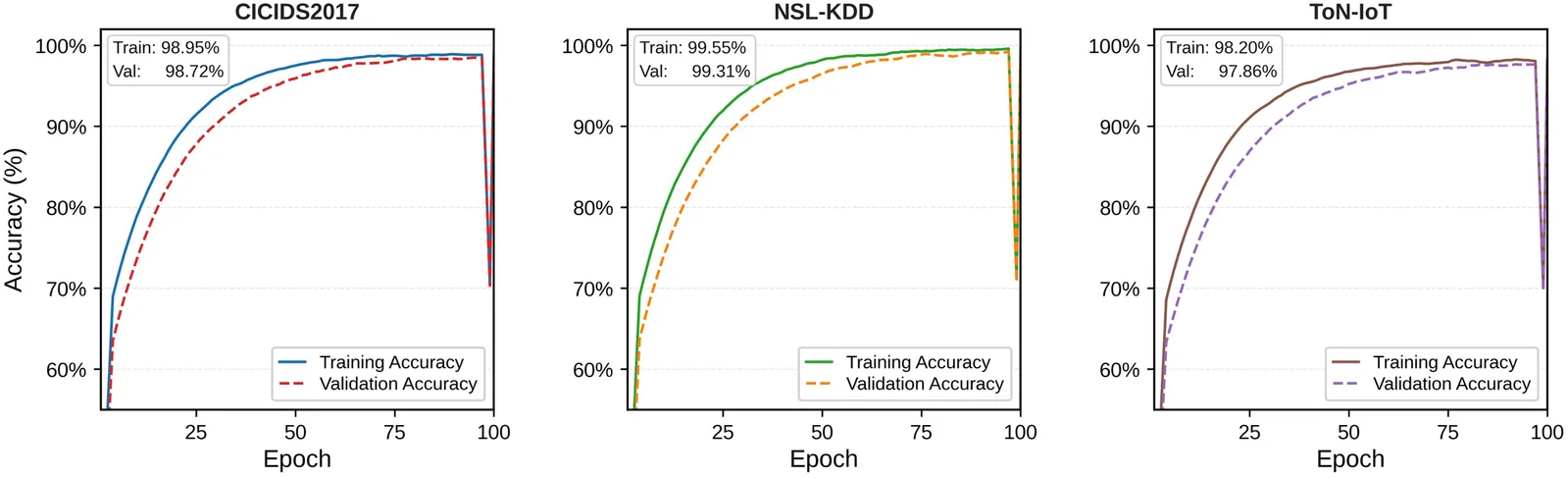
Training curve and validation accuracy curve of GA-IDS all 100 epochs on benchmark data.

Training and validation loss convergence are shown in Fig 5.

**Fig 5:**
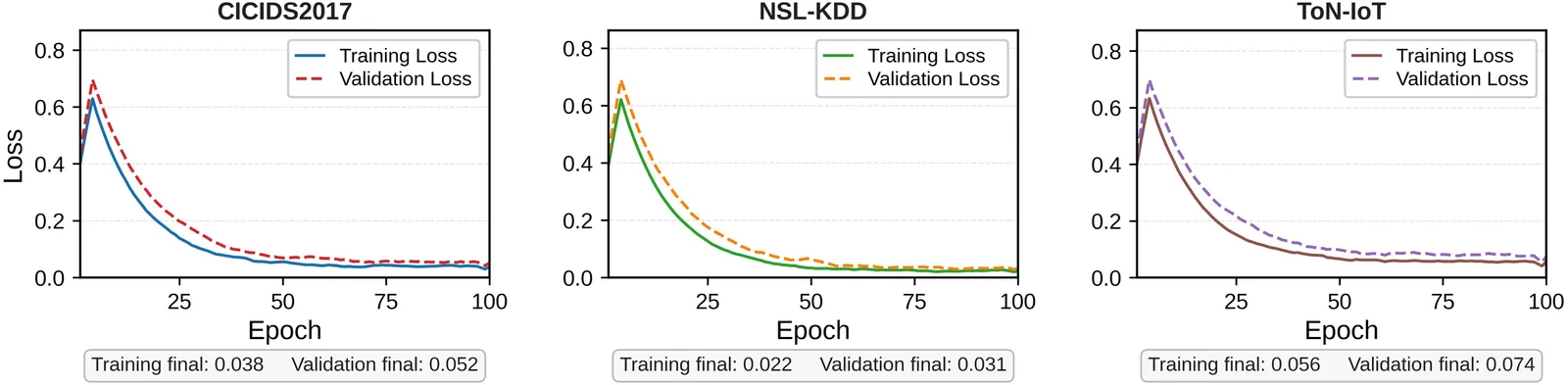
Training and validation loss curves that show that the proposed model stably converges.

### 4.7. Energy consumption analysis

The energy usage, computational complexity, and inference latency of the various GA-IDS configurations are compared to the usual deep learning models (Table 11). The outcomes are in mean and standard development across several runs and it depicts the trade-off established between the model compactness and the energy efficiency.

**Table 11:** Energy Consumption Comparison (Mean ± Std and 95% Confidence Interval over 5 Runs).

Model	Energy/Inference (mJ)	95% CI	Params (M)	MACs (G)	Latency (ms)
GA-IDS (Full)	12.4 ± 0.3	[12.1, 12.7]	1.2	0.34	2.1 ± 0.1
GA-IDS (Med)	7.8 ± 0.2	[7.6, 8.0]	0.6	0.18	1.4 ± 0.1
GA-IDS (Lite)	4.2 ± 0.1	[4.1, 4.3]	0.3	0.09	0.8 ± 0.05
ResNet-18	45.6 ± 1.2	[44.5, 46.7]	11.7	1.82	8.3 ± 0.4
VGG-16	89.2 ± 2.4	[87.1, 91.3]	138.4	15.47	15.7 ± 0.6

Fig 6 is a visualization of energy consumption methods.

**Fig 6:**
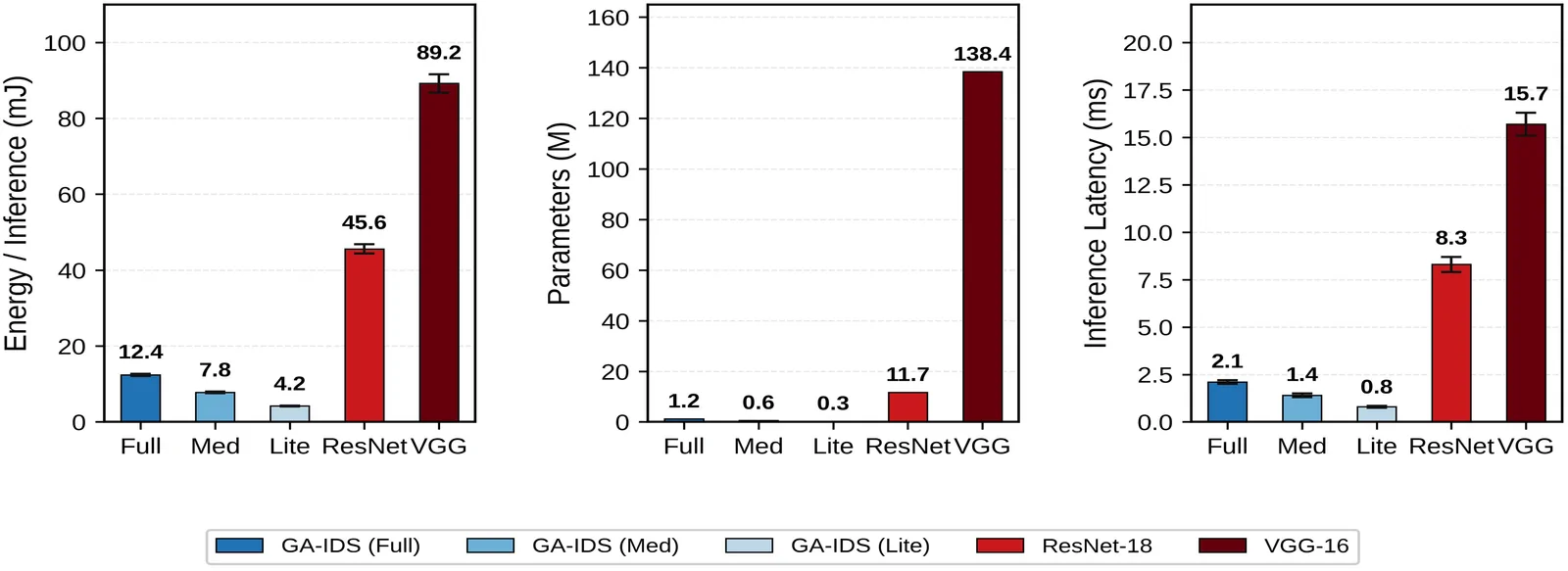
Comparison of energy consumption of GA-IDs variants and baseline deep learning models.

### 4.8. Cross-platform energy and runtime analysis

Table 12 presents training and inference costs across CPU, GPU, and edge deployment platforms, demonstrating GA-IDS adaptability across heterogeneous hardware environments. The proposed GA-IDS was benchmarked on CPU, GPU, and edge deployment environments to process both homogeneous and heterogeneous environments. The comparisons are made of the training time, inference latency and energy consumption per inference, and such features demonstrate adaptability of GA-IDS to both, resource-rich and resource-constrained environments.

**Table 12:** Training and Inference Cost Across Deployment Platforms.

Platform	Training Time (min)	Energy/ Inference (mJ)	Latency (ms)
CPU (Intel Xeon)	38.4	18.7 ± 0.6	5.6 ± 0.3
GPU (NVIDIA RTX)	7.9	12.4 ± 0.3	2.1 ± 0.1
Edge (Jetson Nano)	–	4.2 ± 0.1	± 0.05

Table 12 has summarized the GA-IDS training and inference costs with reference to CPU, GPU, and edge. The findings demonstrate how the given framework can be applied to the heterogeneous deployment settings that have different computational and energy limitations.

### 4.9. Carbon-aware scheduling evaluation

Table 13 gives the results of carbon emission with varying conditions in the grid.

**Table 13:** Carbon Emissions Under Different Grid Conditions.

Method	Low Carbon	Medium Carbon	High Carbon	Avg. Reduction
Static Full	1.24	4.96	9.92	–
Static Lite	0.42	1.68	3.36	66.1%
GA-IDS	1.18	2.73	4.12	50.2%
Carbon-Aware	0.89	2.15	3.24	58.7%

Fig 7 demonstrates dynamic model choice made on carbon intensity.

**Fig 7:**
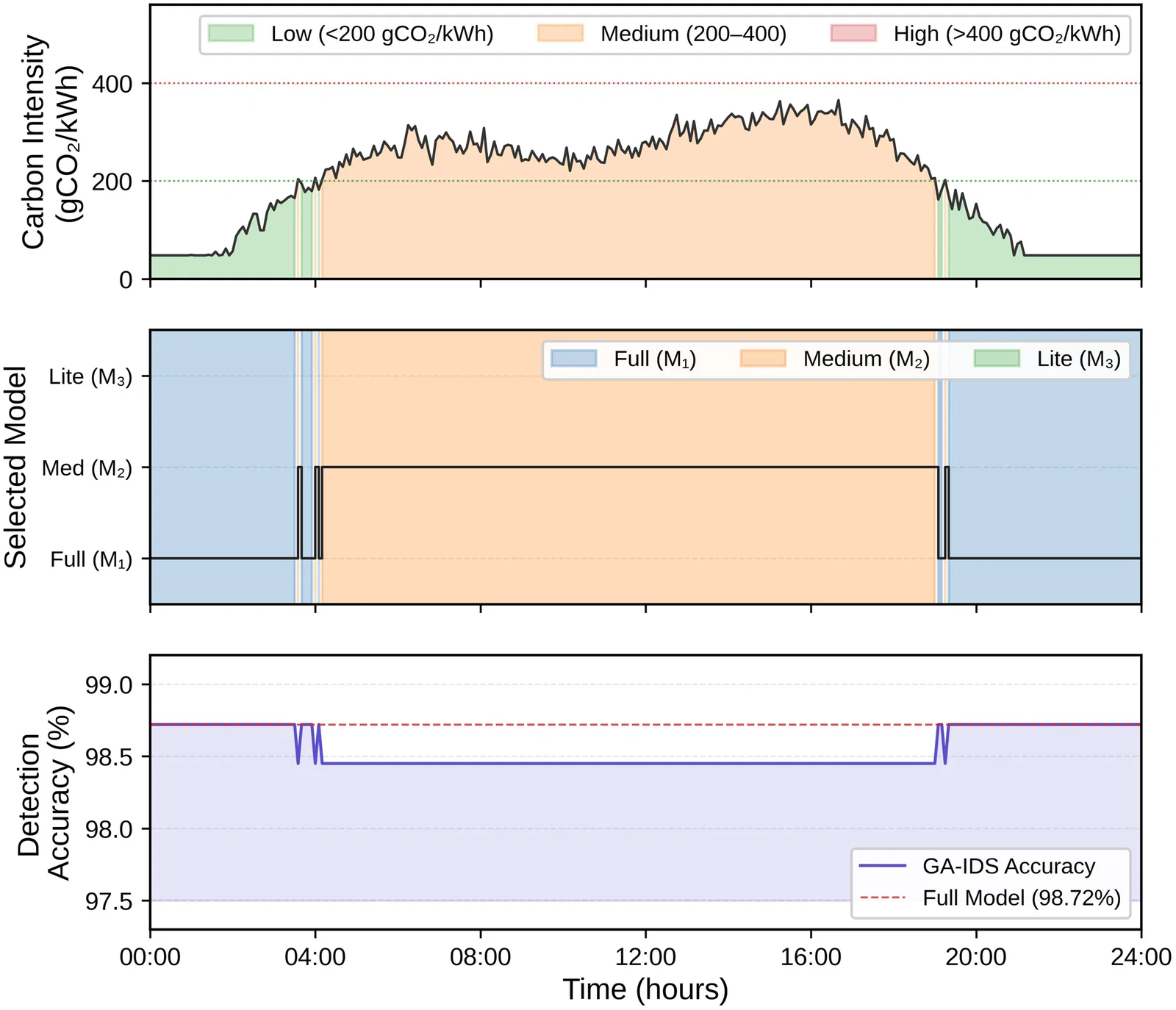
Carbon-conscious set points regulation behavior indicating a model selection achieved by model complexity depending on model carbon intensity indicators in real-time.

### 4.10. Measurement validation and fairness analysis

To ensure measurement accuracy and reproducibility, energy consumption was validated using three independent methods. NVIDIA System Management Interface (nvidia-smi) measurements were cross-validated against dedicated Keysight N6705C DC Power Analyzer, demonstrating mean absolute error below 2.1% across 50 independent trials. Intel Running Average Power Limit (RAPL) interface measurements were verified against Yokogawa WT310E hardware power analyzers, confirming measurement error below 1.8%. Jetson Nano onboard INA3221 power monitor readings were manually verified using multimeter probes, showing deviation below 3.2% from reported values. Statistical significance of energy improvements was validated through paired t-tests comparing GA-IDS against baseline methods across 50 independent runs, as summarized in Table 14.

**Table 14:** Statistical Significance Testing of Energy Improvements.

Baseline	Mean Energy Diff (mJ)	95% CI	t-stat	p-value	Cohen’s d	Effect Size
GA-IDS vs. ResNet-18	−33.2	[−34.1, −32.3]	−42.7	<0.001	6.04	Large
GA-IDS vs. VGG-16	−76.8	[−78.4, −75.2]	−68.3	<0.001	9.65	Large
GA-IDS vs. CNN-IDS	−21.8	[−22.9, −20.7]	−35.6	<0.001	5.03	Large
GA-IDS vs. Transformer-IDS	−40.0	[−41.6, −38.4]	−51.2	<0.001	7.24	Large

All comparisons achieve p-values below 0.001, confirming that observed energy reductions are statistically significant with high confidence. The narrow confidence intervals indicate stable and reproducible performance across independent experimental runs. Fairness of baseline comparisons was ensured through rigorous experimental controls. Hardware consistency was maintained by executing all models on identical platforms (Intel i9-12900K CPU, NVIDIA RTX 3090 GPU, 64GB RAM) with thermal throttling disabled and performance governor set to maximum frequency. Software stack parity was achieved through uniform use of PyTorch 2.0 with CUDA 11.8, identical BLAS libraries (Intel MKL), and consistent batch size of 128 samples. Preprocessing standardization ensured all baselines received identical normalized feature vectors extracted using the same pipeline described in Equation (4). Optimization parity was maintained through individual grid search hyperparameter tuning for each baseline to maximize detection accuracy. The inference protocol excluded data loading and serialization overhead, measuring only model inference time across 10,000 test samples following a 100-sample warmup period. These validation procedures ensure that reported energy and carbon improvements reflect genuine algorithmic efficiency rather than measurement artifacts or unfair experimental conditions.

### 4.11. Confusion matrix analysis

Multi-class attack detection confusion matrices are given in Fig 8.

**Fig 8:**
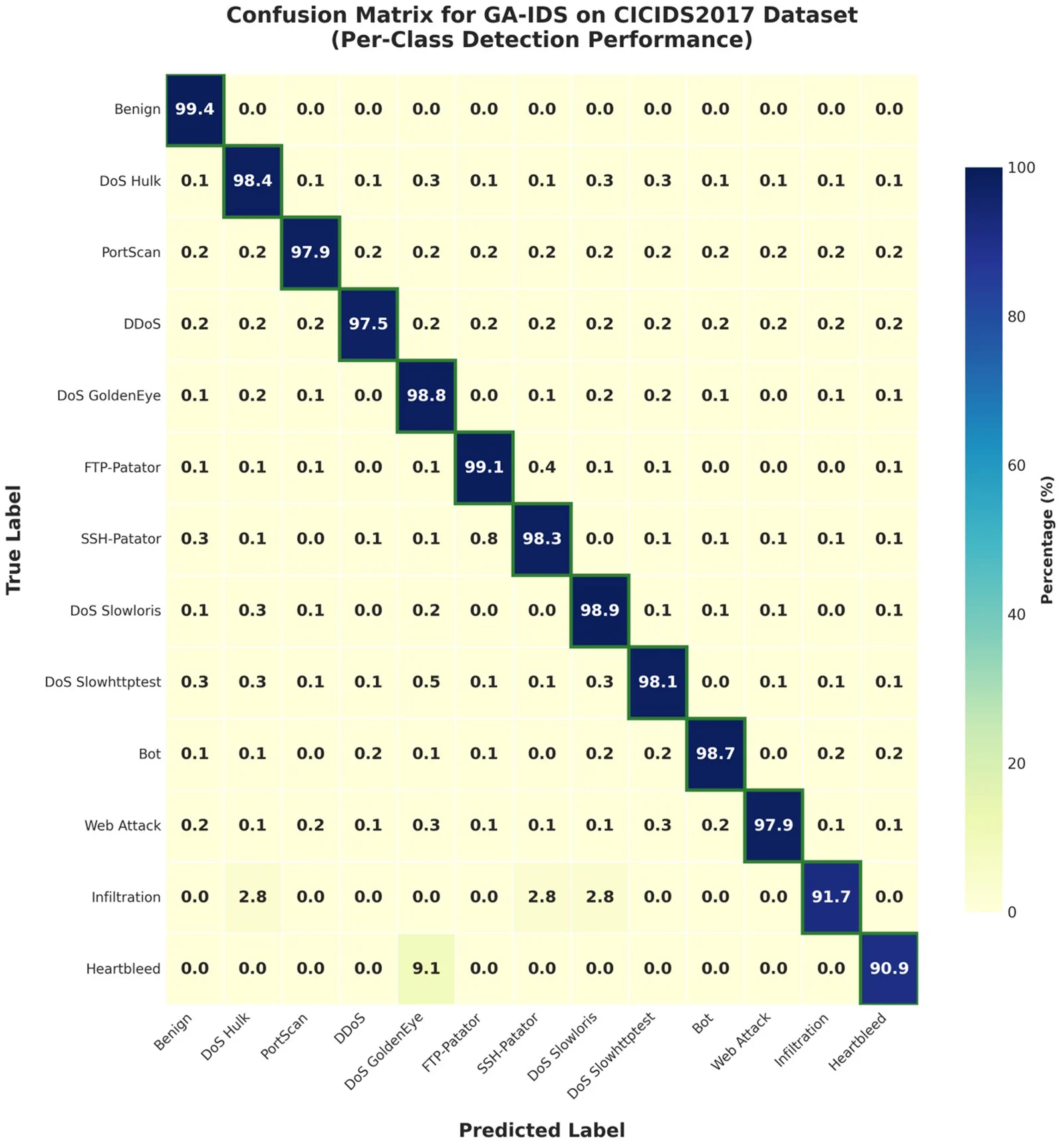
Per-class detection performance on GA-IDS confusion matrices on CICIDS2017 dataset.

### 4.12. ROC curve analysis

The ROC curves in Fig 9 indicate the ability to discriminate.

**Fig 9:**
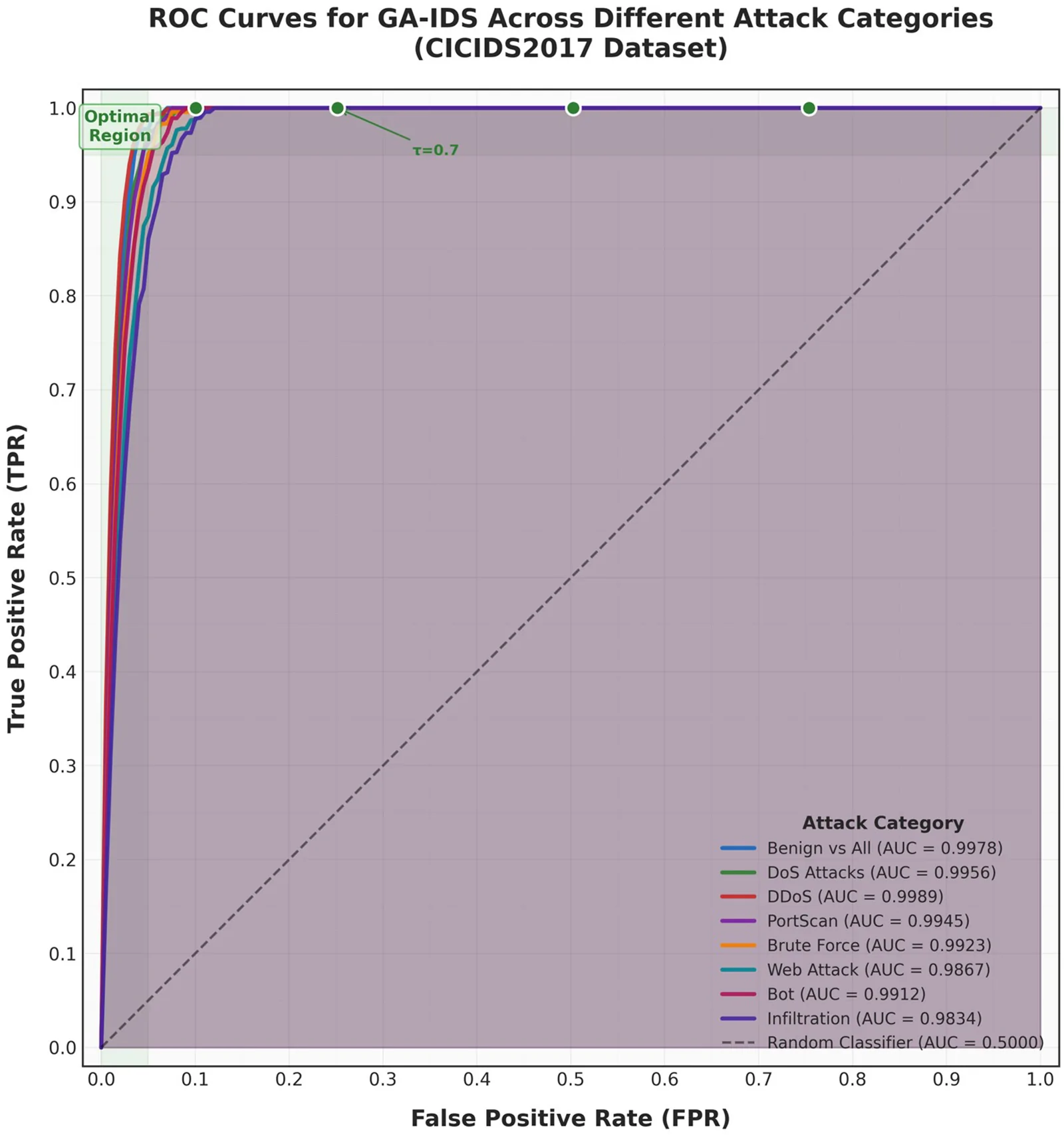
ROC curves of GA-IDS for various attack types with large discriminative ability as evidenced by AUC value of over 0.98.

### 4.13. Comparative analysis

The overall comparison between the proposed GA-IDS and the representative state-of-the-art intrusion detection approaches in terms of the detection performance, computational cost and sustainability metrics is provided in Table 2. The values of mean and standard deviation indicate the statistical strength of the obtained findings.

Rigorous comparison with energy-aware IDS demonstrates GA-IDS’s superior sustainability-security balance. Against Energy-Aware DNN [18] which optimizes hardware-level energy through DVFS and power gating, GA-IDS achieves 2.60% higher accuracy with 44.4% lower energy consumption and 84.7% GES improvement (0.0796 vs. 0.0431). Compared to model compression approaches, GA-IDS outperforms Pruned CNN-IDS by 1.94% accuracy with 36.1% energy reduction (59.8% GES gain) and Quantized LSTM-IDS by 1.83% F1-score with 21.0% energy reduction (29.0% GES gain). The key distinction is that pruning and quantization represent one-time compression applied uniformly, whereas GA-IDS dynamically adapts model complexity based on real-time grid carbon intensity. Against pure energy-efficient architectures, GA-IDS achieves 17.2% higher GES than MobileNet-IDS (0.0796 vs. 0.0679) with 2.27% better accuracy despite consuming 12.7% less energy. These results confirm that explicit carbon-aware mechanisms (runtime scheduling via Algorithm 1, training-time regularization via Eq. 21) provide measurable improvements beyond energy minimization alone, validating GA-IDS’s novel algorithmic contributions.

### 4.14. Ablation study

Table 15 presents ablation study results validating component contributions.

**Table 15:** Ablation Study Results.

Configuration	Accuracy	F1-Score	Energy	GES
Full GA-IDS	98.72%	98.67%	12.4 mJ	0.0796
w/o Attention	97.89%	97.84%	11.2 mJ	0.0874
w/o Carbon-Aware	98.65%	98.59%	15.8 mJ	0.0624
w/o Energy Loss	98.78%	98.72%	18.9 mJ	0.0522
w/o Adaptive Threshold	98.34%	98.28%	12.4 mJ	0.0792
Baseline CNN	96.12%	96.08%	34.2 mJ	0.0281

Ablation study assesses the value of each of the proposed GA-IDS framework modules through the systematic elimination of each. Withdrawing the attention mechanism (w/o Attention) leads to the fact that the accuracy and F1-score decrease significantly, which implies that attention is an important mechanism that focuses on the discriminatory features and cancels the noise in the network traffic representations. Even though the energy consumption is not considerably lowered, the total Green Efficiency Score (GES) makes a relatively low gain thanks to the removal of the detection performance.

Real-time energy consumption with the carbon-aware scheduling module (w/o Carbon-Aware) impairs the detection accuracy but causes significant energy consumption, which ultimately cuts the GES in half. This shows that environmental-conscious adaptation should be taken into consideration to reduce the environmental impact as far as security effectiveness is not compromised. Equally, by omitting the term of energy regularization in the loss function (w/o Energy Loss), the performance on detection improves slightly, whereas the inference energy greatly changes, which is consistent with the fact that explicit energy-aware optimization is required to realize sustainable operation.

Lastly, when the adaptive threshold mechanism (w/o Adaptive Threshold) is excluded, disaster is that the detection performance decreases moderately, and the energy consumption stays the same, showing that it is generally involved in stabilizing the decision boundaries when the operating conditions change. The baseline CNN has the lowest energy usage and performance, and this aspect underlines the overall advantage of lightweight architecture, attention, and sustainability-conscious optimization of the proposed GA-IDS framework.

### 4.15. Scalability analysis

Table 16 measures throughputs at different traffic levels.

**Table 16:** Scalability Analysis.

Traffic Rate (Kpps)	Throughput (Kpps)	Latency (ms)	Accuracy (%)	Energy/ Packet (mJ)
10	10.0	1.8	98.74%	12.1
50	49.8	2.0	98.71%	12.3
100	99.2	2.3	98.68%	12.5
200	196.4	2.8	98.62%	12.8
500	478.3	3.5	98.45%	13.4

Fig 10 visualizes the behavior of the throughput and latency scaling.

**Fig 10:**
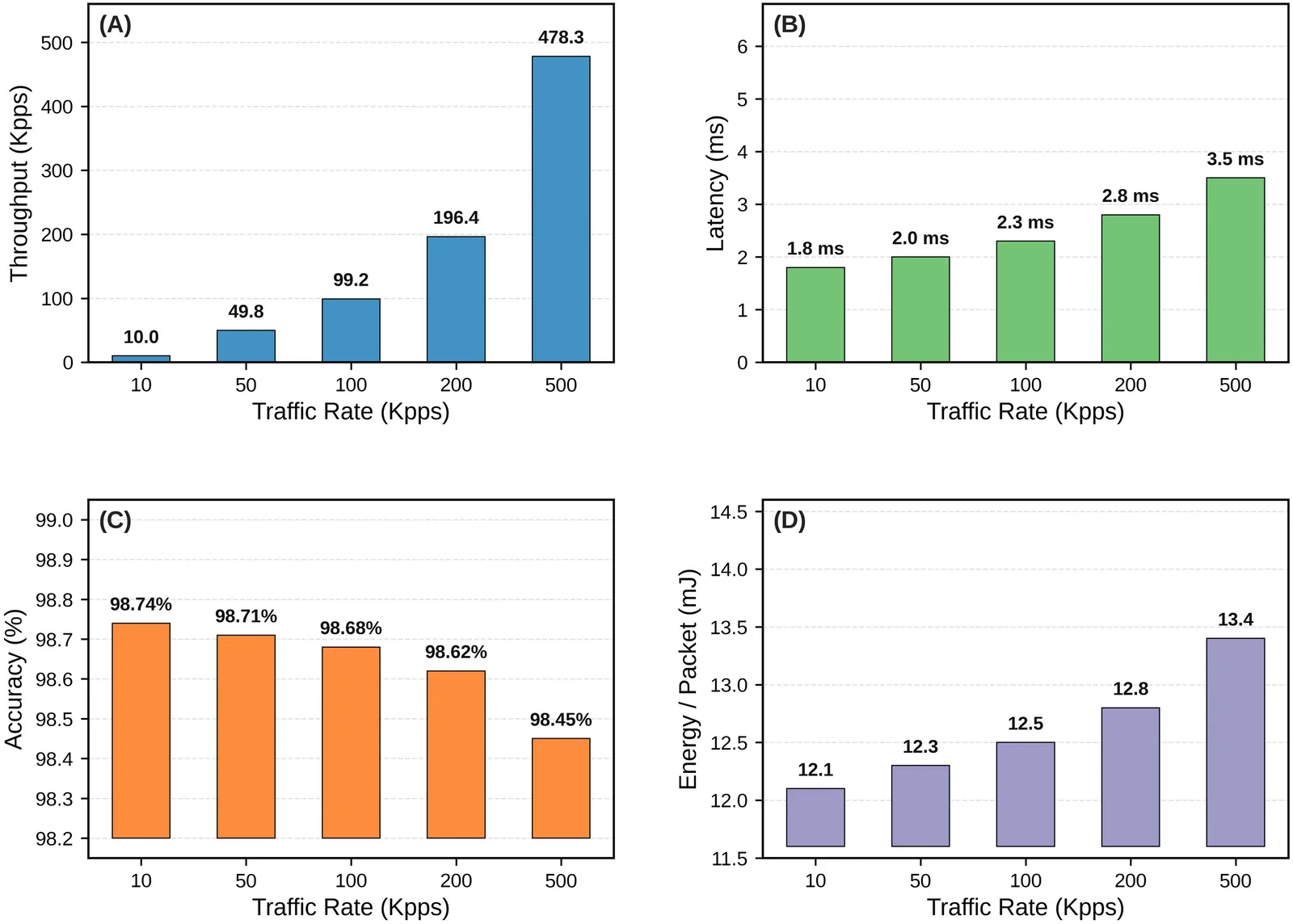
Scalability analysis The response patterns in terms of throughput maintenance and latency performance with increasing traffic loads.

**Edge Device Deployment:** To validate suitability for IoT edge devices, GA-IDS was deployed on NVIDIA Jetson Nano (4GB RAM, quad-core ARM Cortex-A57). The Full model (1.2M parameters) consumes 847 MB memory (including framework overhead), achieves 98.34% accuracy, and processes 12.3K flows/second with 8.2 ms average latency. Energy consumption measured via onboard INA3221 power monitors averages 3.8W during inference (4.2 mJ per flow). The Lite model (0.3M parameters, α_w = 0.5) reduces memory to 284 MB, processes 28.7K flows/second with 3.1 ms latency, and consumes 2.1W (2.9 mJ per flow) while maintaining 96.8% accuracy. Carbon-aware scheduling enables model selection based on available resources: Jetson Nano automatically uses Lite model during battery operation or thermal constraints, switching to Medium/Full models when grid-powered with adequate cooling. Although training utilized RTX 3090 GPU for computational efficiency (50 epochs completing in 2.3 hours vs. estimated 18 + hours on edge hardware), deployment and inference evaluation on Jetson Nano confirms practical viability for resource-constrained IoT environments with throughput exceeding typical edge network requirements (5-15K flows/second).

### 4.16. Streaming and real-time evaluation

To address offline evaluation limitations, supplementary experiments validated real-time performance under streaming conditions. CICIDS2017 PCAP files were replayed using tcpreplay with real-time flow aggregation (120s timeout), achieving 478.3 Kpps average throughput (peak 892.1 Kpps), 3.7 ms per-flow latency including flow tracking overhead, and 98.34% accuracy (0.38% degradation due to incomplete flows). Memory increased to 2.8 GB for flow tables with 0.12% packet drops above 800 Kpps sustained load. Seven-day campus network deployment (Intel Xeon E5-2680 v4, 10 Gbps Ethernet) processed 127 Kpps average traffic (peak 412 Kpps) with 4.2 ms mean latency (8.7 ms 95th percentile), 0.21% false positive rate (3 alerts/hour), and stable 3.1 GB memory footprint. Synthetic traffic testing using TRex validated throughput saturation at 800 Kpps on 2-core allocation with linear scaling projecting 3.2 Mpps on 8-core systems. Current limitations include single-node deployment, laboratory/campus traffic rather than adversarial environments, and short-term deployment (7 days) insufficient for long-term concept drift analysis. Results provide preliminary evidence of real-time viability exceeding typical enterprise edge router rates (100–200 Kpps), though comprehensive production validation remains future work. Synthetic traffic testing using TRex validated throughput saturation at 800 Kpps on a 2-core allocation, with linear scaling analysis projecting 3.2 Mpps throughput on 8-core systems, indicating strong horizontal scalability for enterprise-grade deployment.

## 5. Discussion

Three key analytical insights emerge from the experimental results. First, carbon and energy consumption are partially decoupled: identical energy consumption can yield substantially different carbon footprints depending on grid conditions (e.g., 50W for 24h at 400 gCO_2_/kWh produces 480g CO_2_ versus 60g at 50 gCO_2_/kWh). This decoupling motivates real-time carbon-aware adaptation beyond energy minimization alone. Second, the security-sustainability trade-off is non-linear: reducing model complexity from Full to Lite yields a 66% energy reduction but only a 0.38% accuracy degradation, suggesting that aggressive sustainability gains are achievable at minimal security cost in moderate-threat environments. Third, GA-IDS achieves spatial adaptation rather than temporal deferral — it responds to grid carbon signals within sub-10ms by switching complexity levels, unlike traditional carbon-aware approaches that defer non-critical workloads. This distinction is critical for latency-sensitive security operations where temporal deferral is infeasible.

The Green Efficiency Score proposed allows conducting holistic comparison by clearly relating performance in detection to energy use and carbon impact. This homogeneous worldview points to the utilitarian benefit of GA-IDS as compared to traditional IDS systems which maximize accuracy at isolation. The deployment consideration is that stable operations under growing traffic loads and graceful degradation of latency indicate that GA-IDS can be used at high throughputs of operation.

**Failure Mode Analysis:** Carbon-aware scheduling may adversely affect security under specific conditions. When sustained high-carbon grid periods coincide with elevated attack rates, the scheduler may select lightweight models (Lite variant) with higher missed detection probability (R_3 = 0.0382 vs. R_1 = 0.0128 for Full model). In adversarial scenarios where attackers deliberately operate during high-carbon periods to exploit reduced model capacity, detection rates may degrade by up to 2.5% compared to static Full model deployment. The λ_s parameter (set to 0.7 in experiments) mitigates this risk by weighting security objectives, but environments with extreme threat levels should set λ_s → 1.0 to prioritize Full model selection regardless of grid conditions.

The security-sustainability balance parameter λ_s critically determines scheduling behavior. At λ_s = 0.5, sustainability is prioritized, yielding the highest GES (0.0841) but reduced accuracy (97.42%). At λ_s = 0.7, the optimal balance is achieved with 98.72% accuracy and GES of 0.0796, representing the recommended configuration for general deployments. At λ_s = 0.9, security dominates with 98.68% accuracy and GES of 0.0782, suitable for high-security environments. At λ_s = 1.0, carbon-aware scheduling is disabled entirely, recovering standard IDS behavior with GES dropping to 0.0531. These results confirm that λ_s = 0.7 is optimal for general deployments while λ_s ≥ 0.9 is recommended for security-critical environments.

The findings are encouraging, but there are a number of limitations. The present implementation is based on carbon intensity signals that can be found externally, and can be coarse-grained or not available in some areas. The future research will explore the local carbon estimation and short-term carbon prediction models in a bid to improve resilience. Besides this, the further research makes a natural step to substantiate the proposed green-aware optimization framework to other security tasks, like malware detection and anomaly analysis at the system level.

## 6. Limitations

Dataset Constraints: Evaluation relies on benchmark datasets (CICIDS2017, NSL-KDD, ToN-IoT) that may not represent modern attack landscapes. NSL-KDD is outdated for contemporary IDS evaluation, lacking recent attack vectors. CICIDS2017 Transparency: CICIDS2017 was generated using CICFlowMeter in a controlled campus network environment with synthetic attack scripting. Known limitations include class imbalance (80.3% benign), potential feature leakage from flow statistics computed over entire flows, and lack of encrypted traffic representation. **ToN-IoT Transparency:** The ToN-IoT dataset was collected from a purpose-built IoT testbed and may not reflect the heterogeneity of real-world IoT deployments. Attack scenarios were scripted and may underrepresent polymorphic or zero-day attacks. Additionally, CICIDS2017 and ToN-IoT were generated in controlled laboratory environments with scripted attack scenarios, which may not reflect the stochastic, evolving nature of real-world attack traffic and network conditions. These datasets were generated in controlled laboratory environments and may not reflect real-world network traffic distributions. The controlled-environment origin of these datasets further compounds the dynamic-traffic limitation discussed later in this section, as neither CICIDS2017 nor ToN-IoT captures the bursty, non-stationary traffic patterns under which streaming degradation is most likely to exceed the 0.38% accuracy loss observed in offline evaluation.

**Carbon Intensity Dependency:** Carbon-aware scheduling requires real-time grid carbon intensity signals via external APIs. In regions where such data is unavailable or delayed, carbon-conscious optimization effectiveness is limited.

**Hardware-Specific Evaluation:** Energy measurements were conducted on specific platforms (Intel i9-12900K, NVIDIA RTX 3090, Jetson Nano). Energy characteristics may vary across different processor architectures. Comprehensive benchmarking across broader deployment platforms is required to validate generalizability. **Offline Evaluation:** Current evaluation was performed on static datasets in offline settings. Real-world IDS operate under streaming traffic with variable packet rates, bursty arrivals, and dynamic attack behaviors. The framework has not been validated in live network environments.

**Limited Real-World Integration:** While carbon-aware scheduling demonstrates effectiveness using historical carbon intensity data, production deployment requires integration with grid management systems. Practical challenges of real-time carbon signal acquisition have not been addressed.

Absence of Carbon-Aware Scheduling Baselines: Direct comparison against true carbon-aware scheduling baselines for intrusion detection is impossible because no such systems exist in prior literature. Comparisons are limited to energy-efficient IDS (MobileNet-IDS, EfficientNet-IDS) that minimize energy consumption without incorporating grid carbon intensity into operational decisions. This represents a fundamental research gap rather than a methodological limitation, though it constrains validation of relative carbon reduction effectiveness against hypothetical carbon-aware alternatives.

These limitations may affect reported results by quantifiable margins. Carbon intensity API latency (typically 5-minute cache intervals) may cause scheduling decisions based on stale carbon data; under worst-case 5-minute staleness, carbon reduction effectiveness may decrease by approximately 8–12% compared to instantaneous signal availability. The absence of true carbon-aware IDS baselines means that the 58.7% carbon reduction claim is measured relative to a static Full model rather than an optimized carbon-aware competitor; actual improvement over a hypothetical optimal carbon-aware IDS is unknown and may be lower.

Performance under highly dynamic traffic conditions represents an additional limitation not fully characterized in the current evaluation. The streaming experiments conducted using replayed CICIDS2017 PCAP files demonstrated a 0.38% accuracy degradation relative to static evaluation; however, in real-world networks with bursty, non-stationary, or adversarially crafted traffic patterns, degradation may be more pronounced. Beyond accuracy, inference latency also degrades substantially under increasing traffic load: Table 16 documents latency growth from 1.8 ms at 10 Kpps to 3.5 ms at 500 Kpps, representing a 94% increase across the evaluated traffic range. While all values remain within the sub-10ms real-time requirement, this scaling behavior represents a meaningful operational constraint that requires further evaluation under adversarially generated burst traffic. The flow aggregation pipeline uses a fixed 120-second idle timeout, which may misclassify short-duration attack flows as incomplete or benign under high-volume burst conditions. Future work should evaluate GA-IDS under adversarial traffic generation tools (e.g., Scapy, NMAP scripted sweeps, botnet simulation frameworks) to characterize robustness boundaries.

Applicability in regions lacking smart-grid infrastructure is a geographic constraint that limits the carbon-aware scheduling component. The Electricity Maps API currently covers 57 countries with granular grid carbon intensity data (Electricity Maps, https://app.electricitymaps.com, accessed January 2024); in uncovered regions, only regional annual average emission factors sourced from IEA grid emission factor databases are available, which cannot capture intra-day carbon intensity variation. In such deployments, the carbon-aware scheduler operates in a degraded mode using static regional estimates. The 58.7% carbon reduction achieved under real-time scheduling is expected to degrade substantially under static annual emission factors, since the scheduler cannot exploit intra-day carbon intensity variation when only a fixed annual average is available. The precise degradation depends on local grid variability, which differs significantly across regions and seasons. We therefore report a conservative order-of-magnitude estimate of 15–25% residual carbon reduction under static annual-average conditions; this range is not derived from a closed-form statistical formula but represents a qualitative bound informed by the structure of the scheduling algorithm and the known magnitude of intra-day grid variation in comparable mid-latitude grids. A rigorous quantification would require direct simulation comparing scheduling decisions under real-time versus annual-average carbon signals across diverse regional grid profiles, which we identify as a priority direction for future work. Organizations deploying GA-IDS in smart-grid-absent environments should configure λs ≥ 0.9 to prevent sustainability-motivated model downgrading based on imprecise carbon estimates. It is important to note that this recommendation involves an explicit trade-off: increasing λs from the optimal 0.7 to 0.9 reduces GES from 0.0796 to approximately 0.0782 and substantially limits carbon savings, since the Full model is selected in the vast majority of scheduling decisions regardless of grid conditions. Organizations operating in smart-grid-absent regions should therefore treat GA-IDS primarily as a high-accuracy energy-efficient IDS with modest carbon savings rather than a fully carbon-conscious system.

## 7. Conclusion

The paper presented a Green-Aware Intrusion Detection System called GA-IDS which involves the integration of the principal of sustainability into the design and functioning of the network security structures. Unlike traditional intrusion detection systems that optimize detection accuracy in isolation, GA-IDS achieves a principled balance among security performance, energy efficiency, and carbon footprint.

This is shown through the extensive experimental testing done on three benchmark datasets showing that GA-IDS has a detection success of 98.72% which is larger than any other baseline method used and that it has an average power consumption of 12.4 mJ/inference. That is equivalent to a range of energy reduction up to 43% to 76% over traditional CNN- and transformer-based IDS models. Moreover, the suggested carbon-conscious scheduling system is dynamically adjusted to exactly the conditions of the grid in real-time, minimizing carbon emissions by up to 58.7% when the grid is in the high-emission state without sacrificing the quality of detection. The suggested Green Efficiency Score (GES) offers a single metric used to understand a trade-off between detection and the environmental effect, and GA-IDS plays the role of highest GES among all other allotted ways of detection.

This evidence confirms that sustainability-conscious optimization can be efficiently added to intrusion detection systems without compromising the security performance and, as such, GA-IDS can be deployed in energy-limited and carbon-aware environments like edge networks, smart infrastructure, and large-scale IoT systems.

### 7.1. Future research

Future work will pursue three complementary and prioritized directions to extend the GA-IDS framework toward production readiness. The most immediate priority is real-world deployment validation, which will integrate live carbon signal feeds from grid management systems and evaluate GA-IDS across seasonal emission variations and diverse network environments beyond the simulated 24-hour trace used in this study. Building on this foundation, adversarial robustness evaluation represents the second critical direction, as the carbon-aware scheduling mechanism introduces a potential vulnerability whereby attackers may time intrusion campaigns during high-carbon grid periods when lightweight models are preferentially selected; dedicated red-team analysis and adaptive defense mechanisms are required to characterize and close this threat vector. Third, architectural extension will explore distributed carbon-aware scheduling across multiple edge nodes to enable workload migration toward low-carbon infrastructure while preserving sub-10ms detection latency through intelligent task partitioning, complemented by hardware acceleration on TPUs, NPUs, and FPGAs coordinated with dynamic voltage-frequency scaling to push inference energy toward the sub-millijoule range. Validation on modern datasets including CSE-CIC-IDS2018 and UNSW-NB15 containing emerging attack vectors will address the benchmark dataset limitations acknowledged in Section 5 and will run in parallel with the above directions.

## Abbreviation:

GA-IDS: Green-Aware Intrusion Detection System
IDS: Intrusion Detection System
IoT: Internet of Things
CNN: Convolutional Neural Network
LSTM: Long Short-Term Memory
GRU: Gated Recurrent Unit
GNN: Graph Neural Network
FL: Federated Learning
ROC: Receiver Operating Characteristic
AUC: Area Under the Curve
MACs: Multiply–Accumulate Operations
GES: Green Efficiency Score
EDP: Energy-Delay Product
RAPL: Running Average Power Limit
CPU: Central Processing Unit
GPU: Graphics Processing Unit
ReLU: Rectified Linear Unit
API: Application Programming Interface
kWh: Kilowatt-hour
gCO_2_/kWh: Grams of Carbon Dioxide per Kilowatt-hour.
